# Supplementary figures and images for: Crosslinking-guided geometry of a complete CXC receptor-chemokine complex and the basis of chemokine subfamily selectivity
Source: PLoS Biol. 2020 Apr 9;18(4):e3000656. doi: 10.1371/journal.pbio.3000656 (PMC7173943; doi:10.1371/journal.pbio.3000656)

Uncropped gels and blots for Fig 3

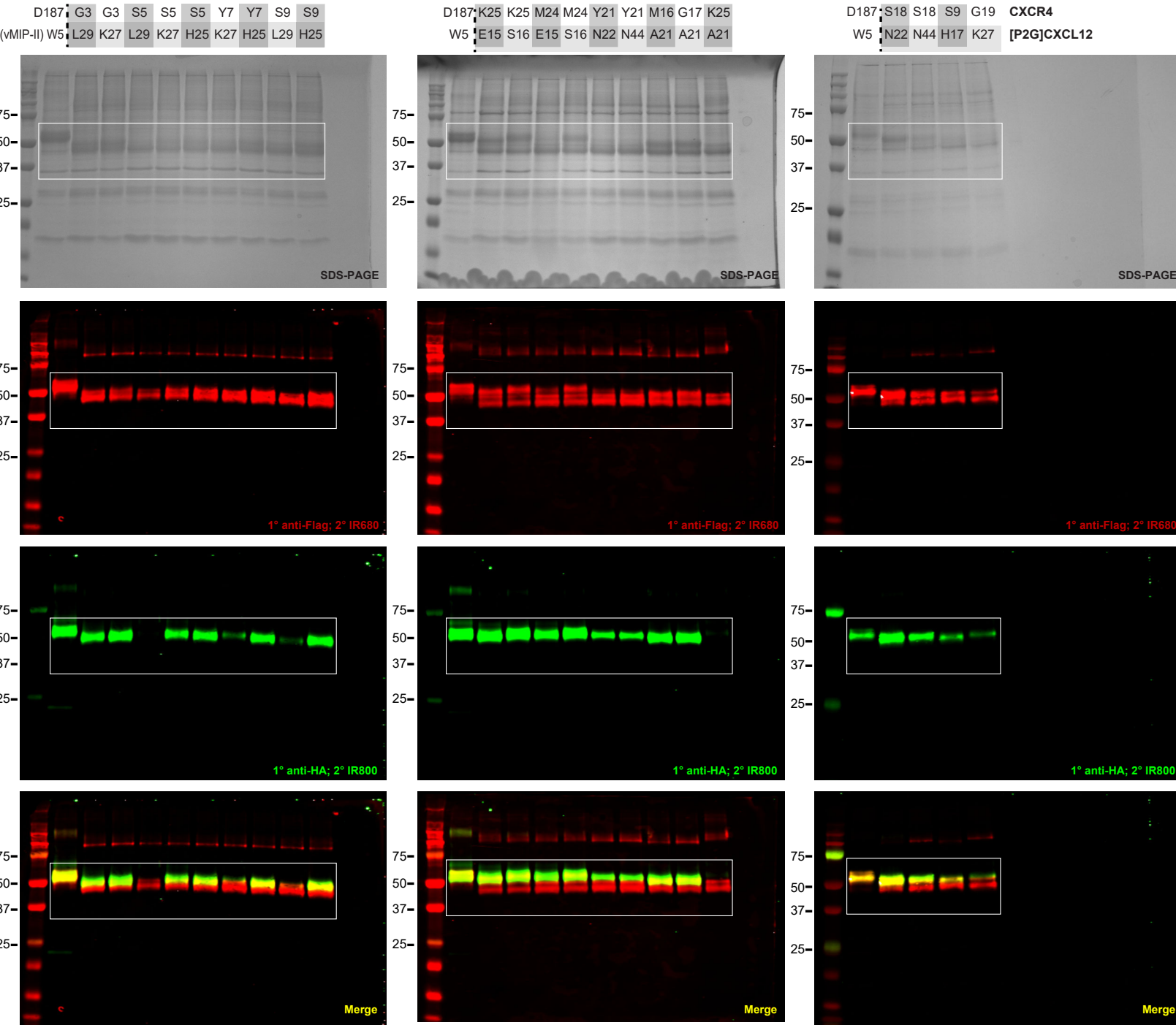

Uncropped blots for S5 Fig

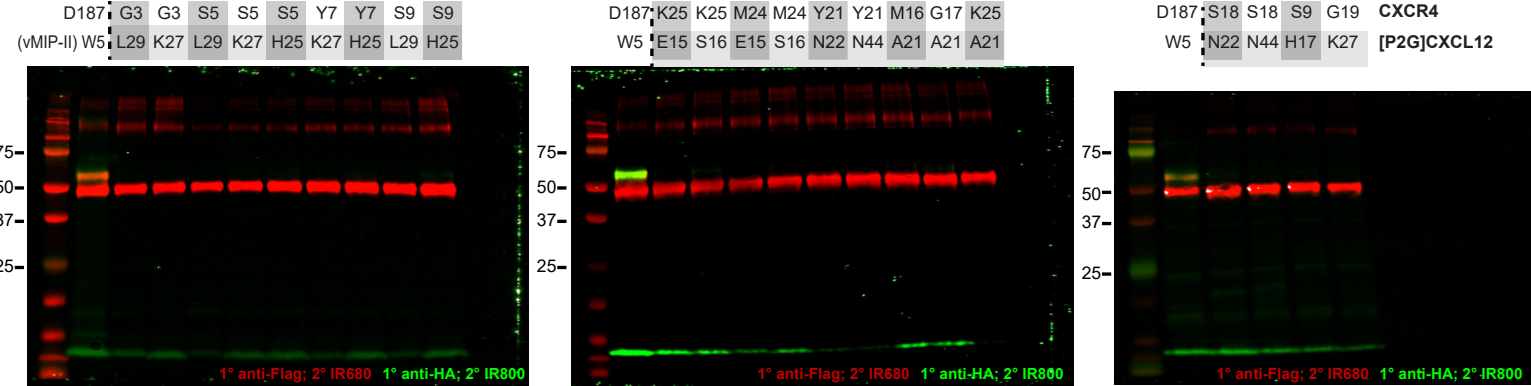

Supplement: S1 Raw Images for Gels and Blots — (PDF) [file pbio.3000656.s001.pdf]

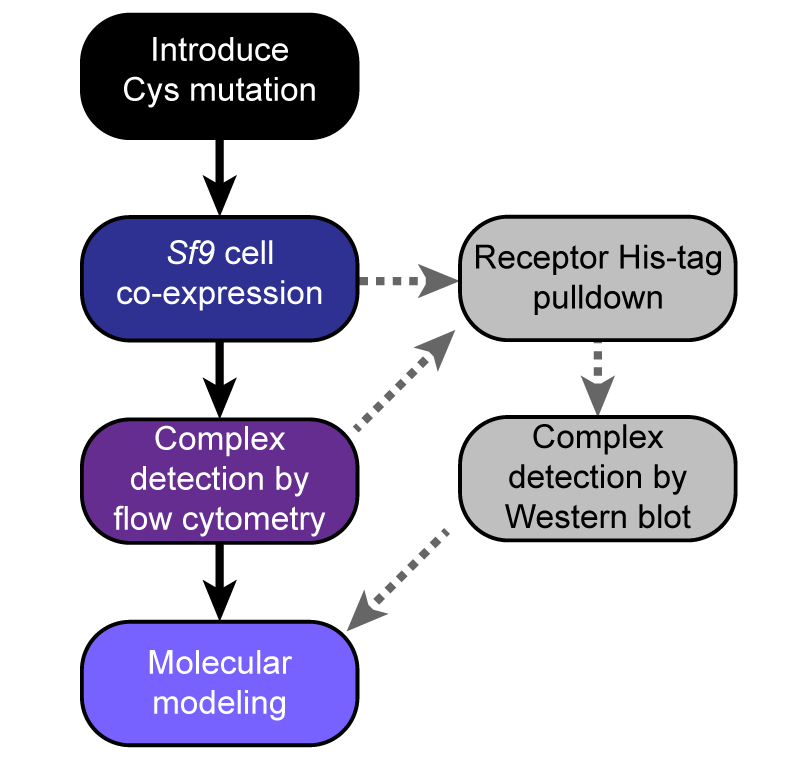

Supplement: S1 Fig — (TIF) [file pbio.3000656.s002.tif]

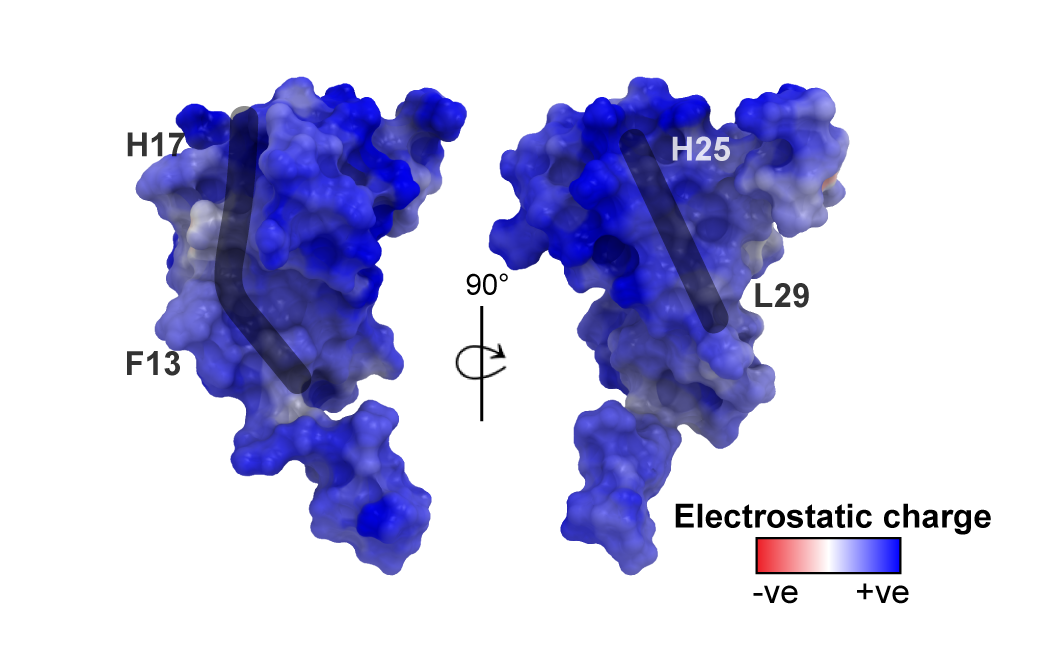

Supplement: S2 Fig — The CXCL12 surface is strongly positively charged (blue). The electrostatic surface was calculated by the Rapid Exact-Boundary ELectrostatics (REBEL) method in ICM [79]. The black strokes highlight the predicted peptide interaction grooves on the CXCL12 surface: one between the N-loop and 40s loop, and another along the β1-strand backbone. (TIF) [file pbio.3000656.s003.tif]

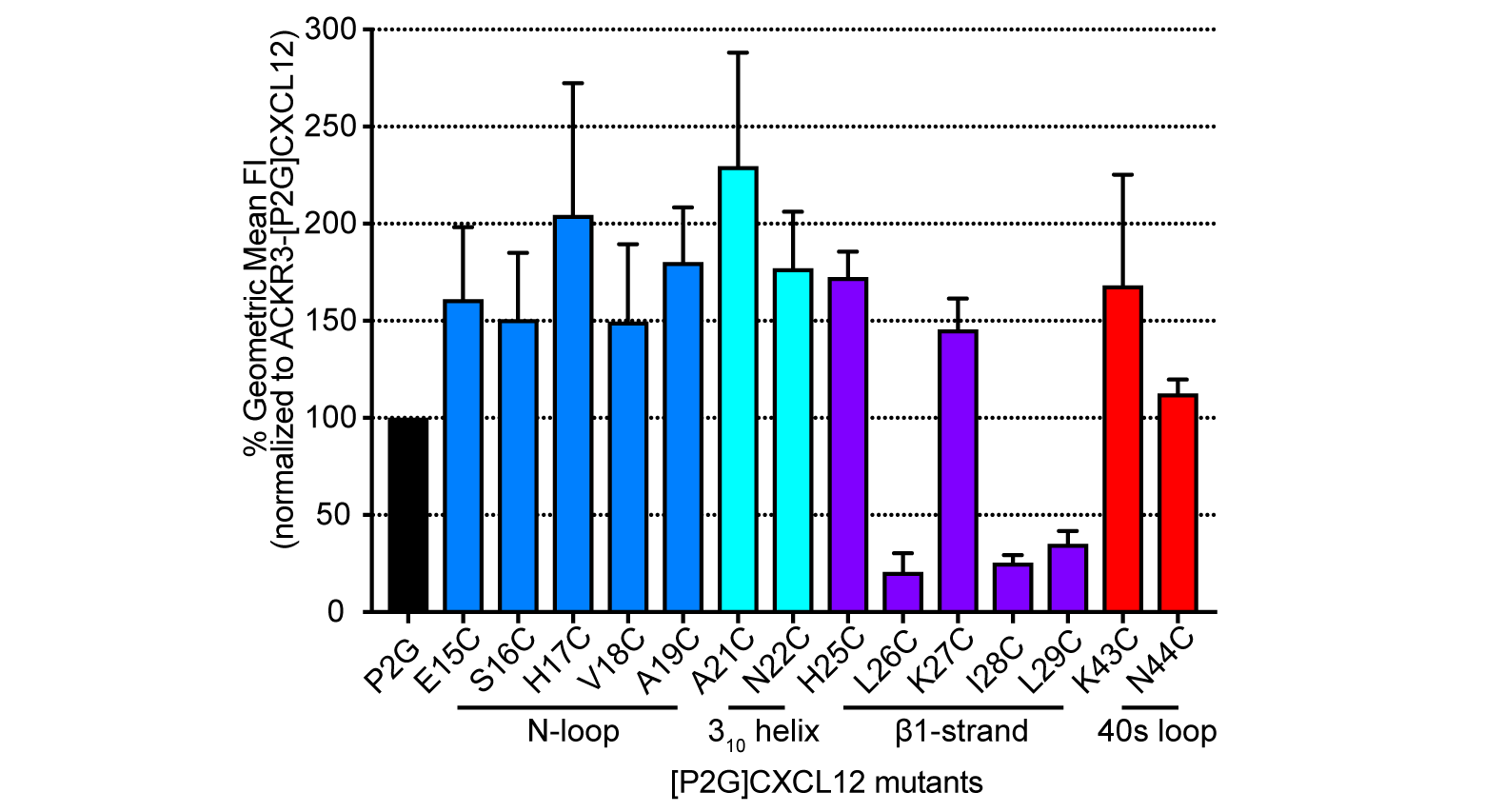

Supplement: S3 Fig — ACKR3 and [P2G]CXCL12 cysteine mutants were co-expressed in Sf9 cells. Due to the slow off-rate of [P2G]CXCL12 with ACKR3, complexes readily detected on the cell surface are a proxy for mutant chemokine folding. All mutants except L26C, I28C, and L29C retain their ability to bind ACKR3. n = 4 independent biological replicates. The mean and SEM are reported for each point. The underlying numerical data for the figure can be found in S1 Data. Sf9, Spodoptera frugiperda. (TIF) [file pbio.3000656.s004.tif]

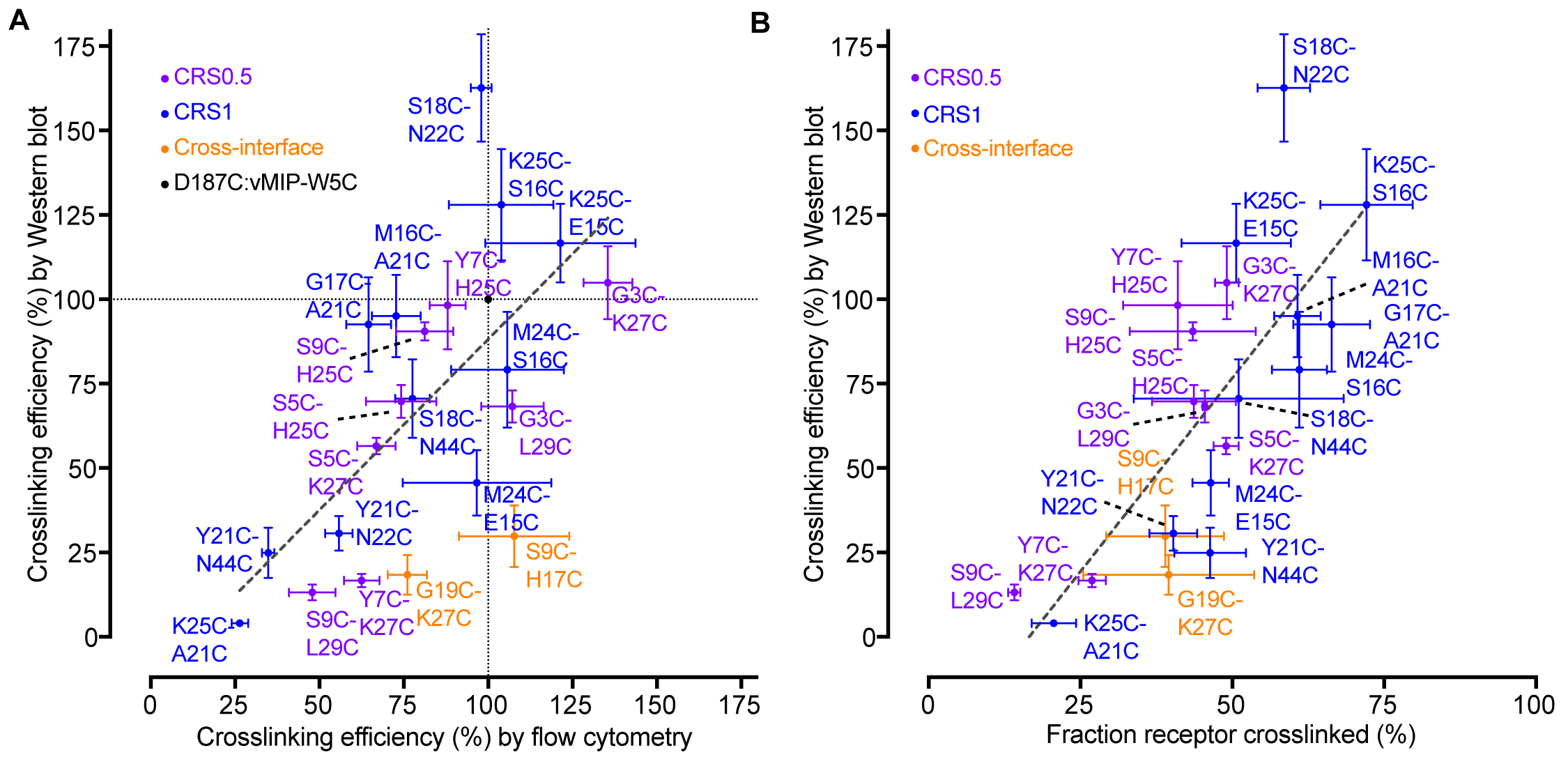

Supplement: S4 Fig — Positive relationship between the detection of the chemokine HA tag on the cell surface and in the pulled down cross-linked samples (A) and between the HA intensity and the fraction cross-linked receptor in the pulled down samples (B). Flow cytometry was used for detection of the chemokine HA tag on the cell surface, whereas Western blot was used for pulled-down protein samples. Data represent mean and SEM of 3 or more independent replicates. The underlying numerical data for each figure panel can be found in S1 Data. (TIF) [file pbio.3000656.s005.tif]

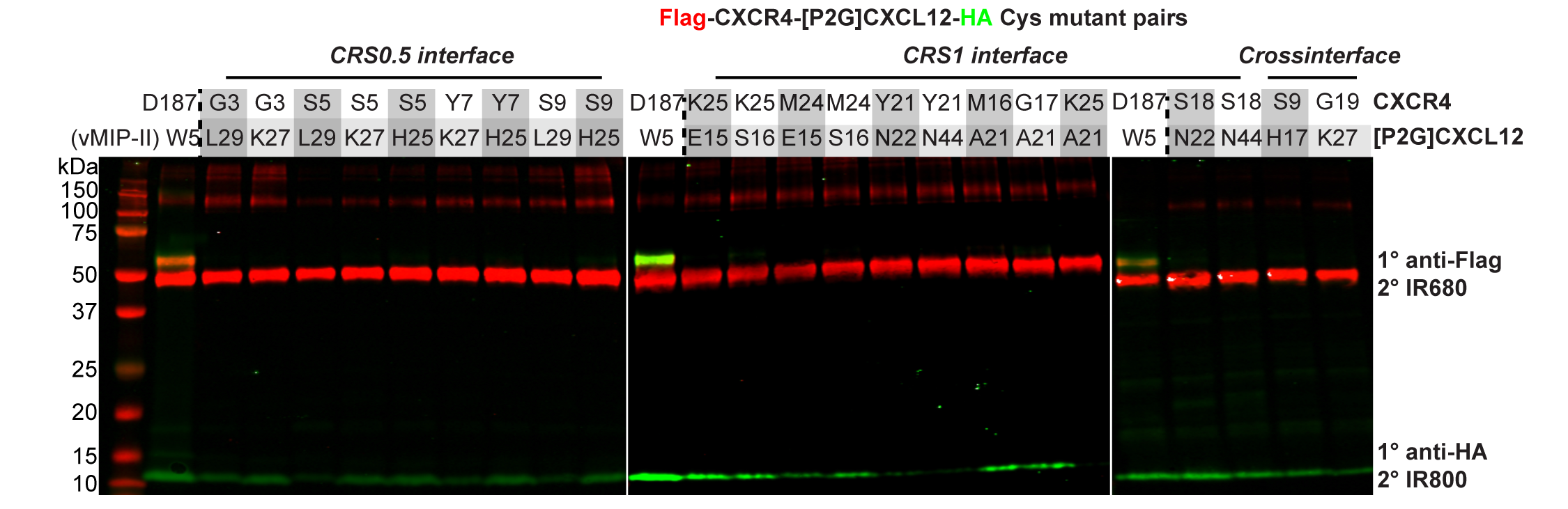

Supplement: S5 Fig — Western blot of pulled down combinations of CXCR4 and [P2G]CXCL12 cysteine mutants in the presence of 100 mM DTT. The Flag-CXCR4-T4L receptor and [P2G]CXCL12-HA chemokine were detected by LI-COR IRDye conjugated secondary antibodies on a single blot (emission wavelength of 680 nm and 800 nm, visualized in red and green, respectively). Emitted fluorescence detected at 800 nm and 680 nm from different bands of the Western blot is indicative of a dissociated receptor-chemokine complex. The figure is representative of n = 3 independent replicates. (TIF) [file pbio.3000656.s006.tif]

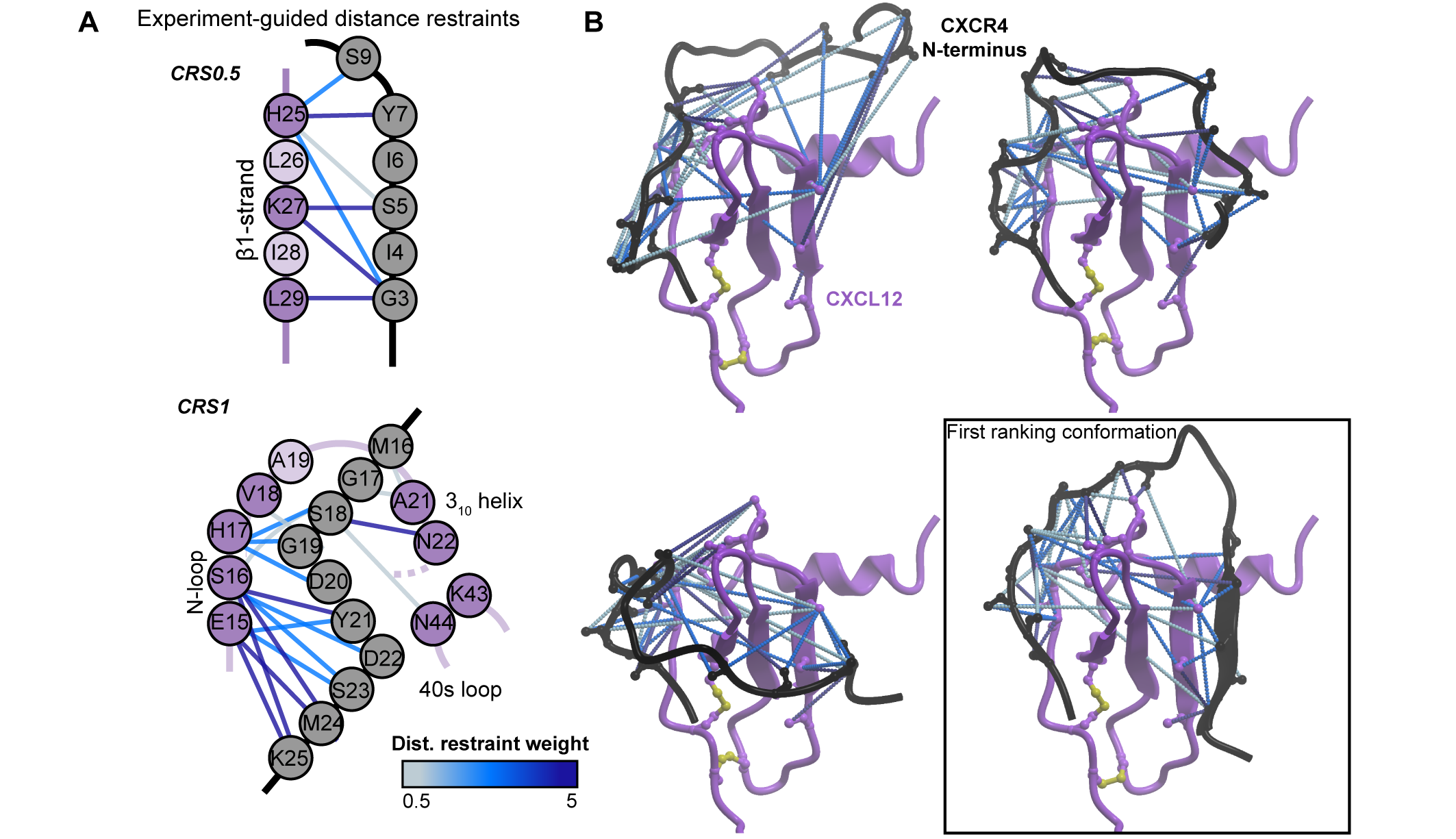

Supplement: S6 Fig — (A) Graphical representation of the experimentally derived local distance restraints imposed during the molecular docking simulations. Cross-interface restraints are not shown. Distance restraints are colored by a gradient of blue according to their experimentally determined strength. (B) The distance restraints are mapped onto 3 randomly selected starting conformations and the top ranked conformation of the receptor N terminus. Distance restraints are shown in dotted lines, colored by a gradient of blue as in panel A. The receptor N terminus and CXCL12 are shown in black and purple ribbon, respectively. The underlying values of the distance restraint weights are found in S4 Table. (TIF) [file pbio.3000656.s007.tif]

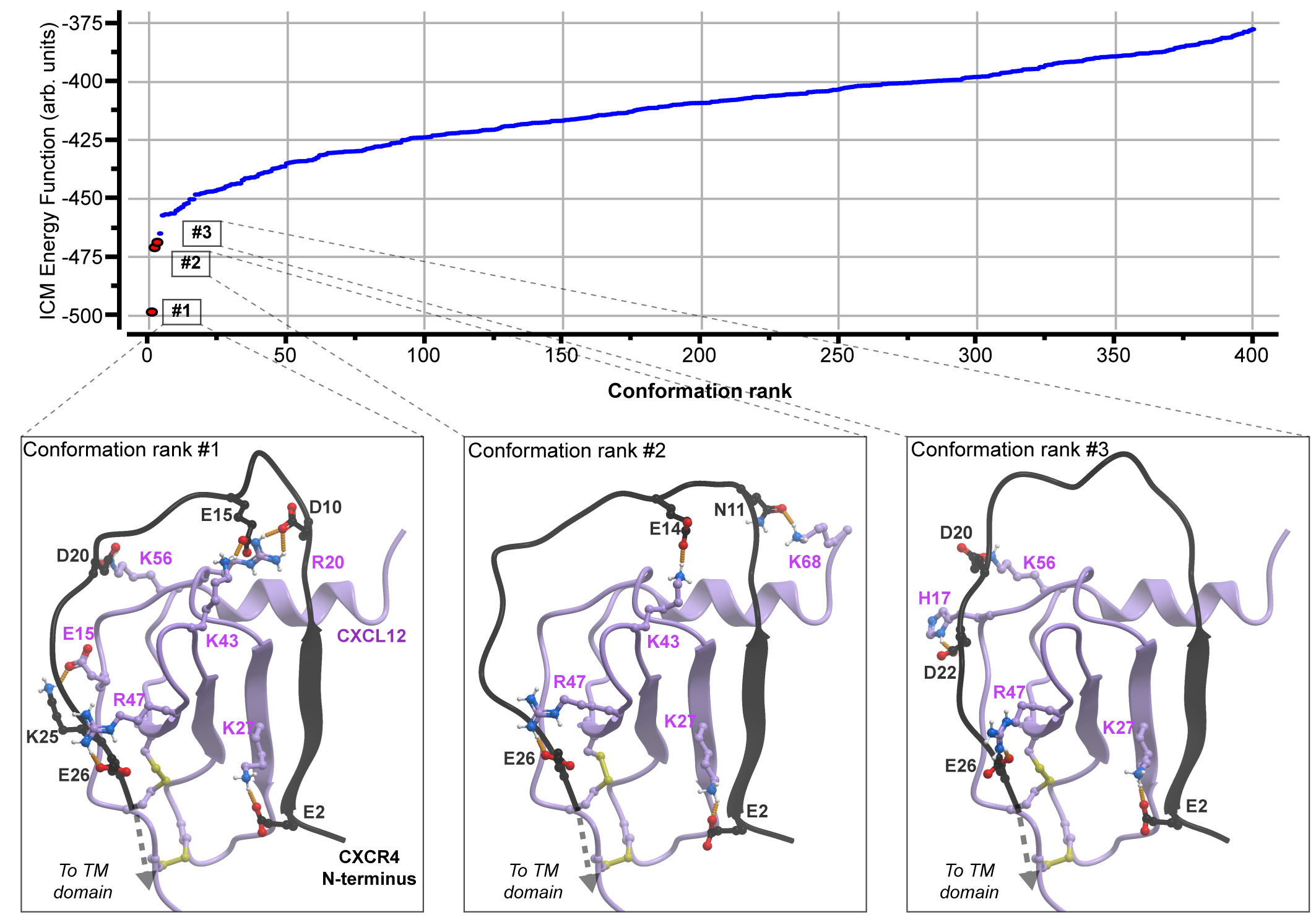

Supplement: S7 Fig — The lowest energy conformations are distinct from the other conformations. The conformational stack was sorted by the energy of the system. Polar and charge interactions are shown in orange dotted lines. The receptor and CXCL12 are shown in black and purple ribbon, respectively. The TM domain is hidden for clarity. The underlying numerical data for the figure can be found in S1 Data. TM, transmembrane. (TIF) [file pbio.3000656.s008.tif]

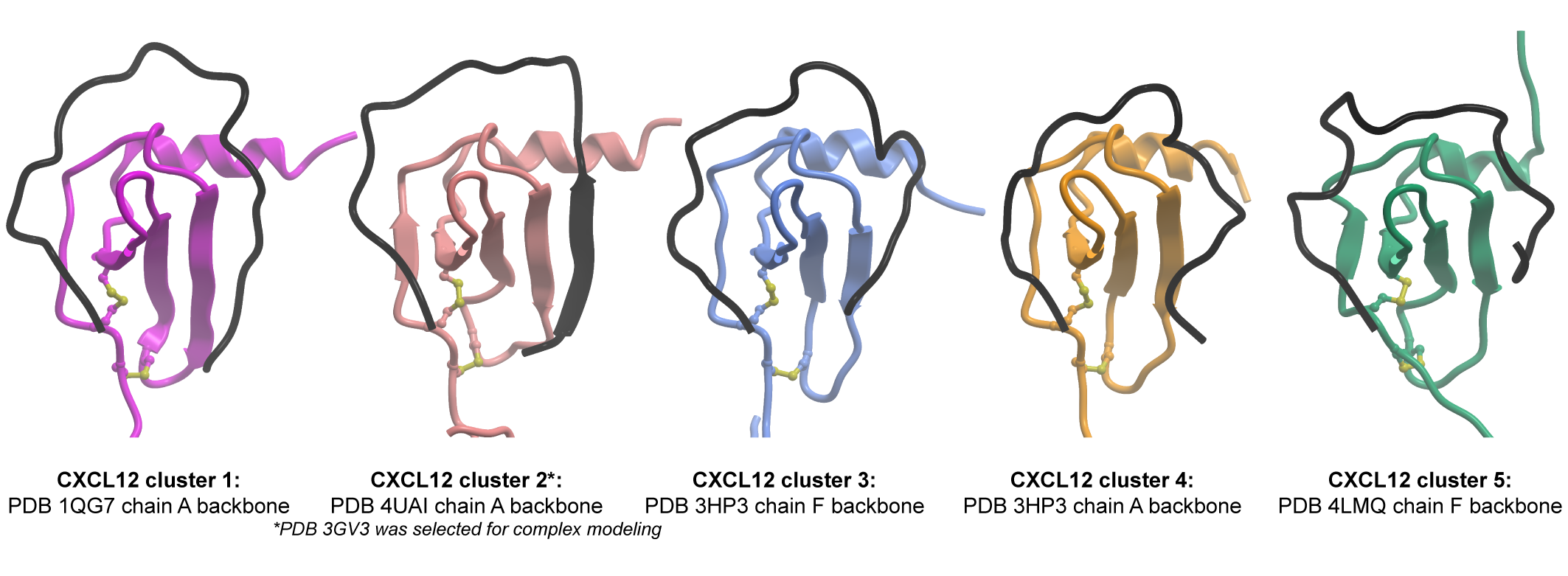

Supplement: S8 Fig — The top-ranking conformation from each respective simulation is shown: in all cases, the receptor N terminus forms an interface with the CXCL12 β1-strand. The CXCL12 conformation PDB 3GV3 from Cluster 2 was selected for full-length complex assembly. The receptor is shown in black, and CXCL12 is colored distinctly in each model. CRS, chemokine recognition site. (TIF) [file pbio.3000656.s009.tif]

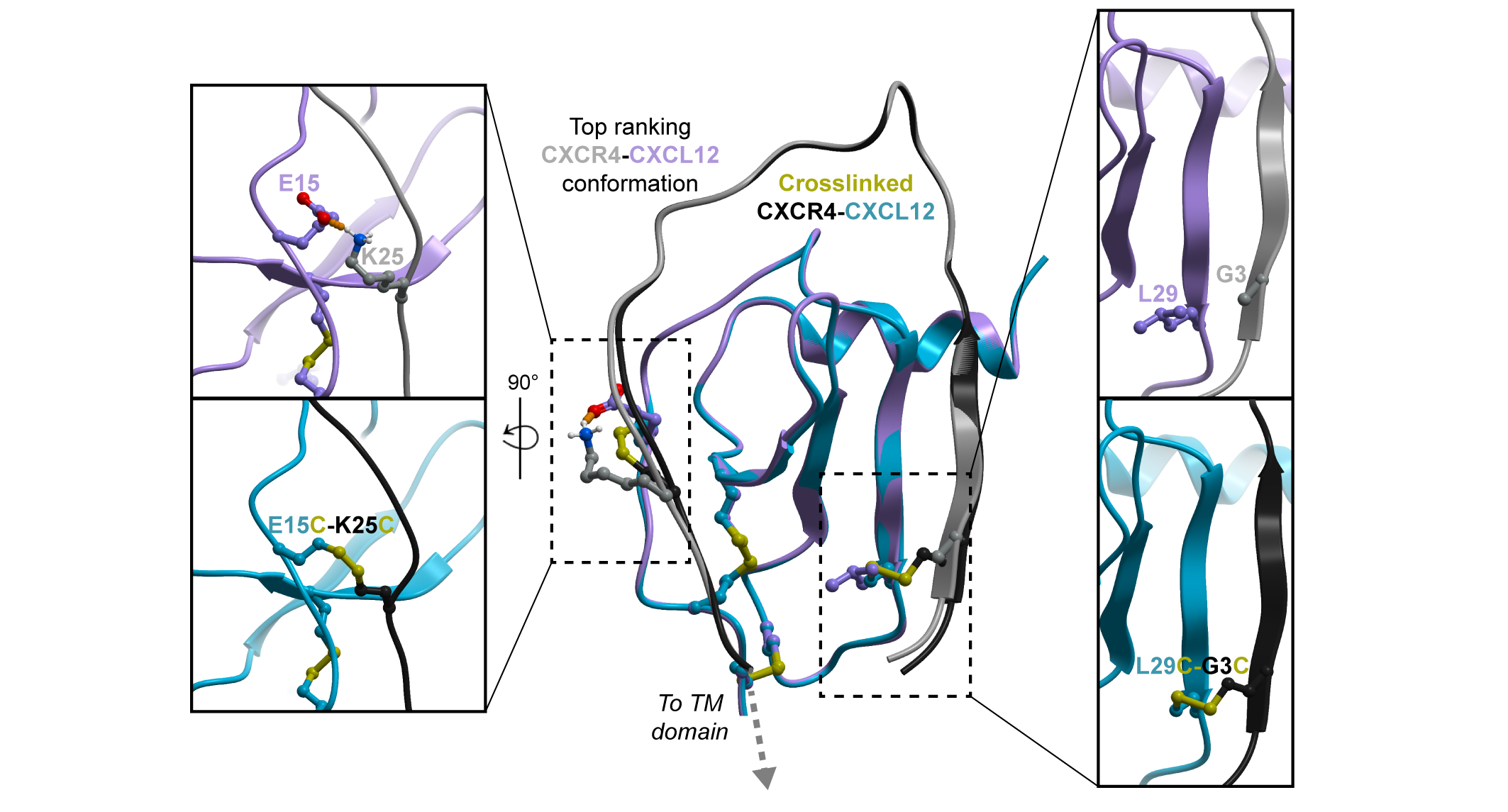

Supplement: S9 Fig — Experimental cross-linking observed between the 2 pairs of CXCR4-CXCL12 residues (E15-K25 and L29-G3) can easily be accommodated structurally in our top-ranking model with minor changes to the conformation of the receptor N terminus. (TIF) [file pbio.3000656.s010.tif]

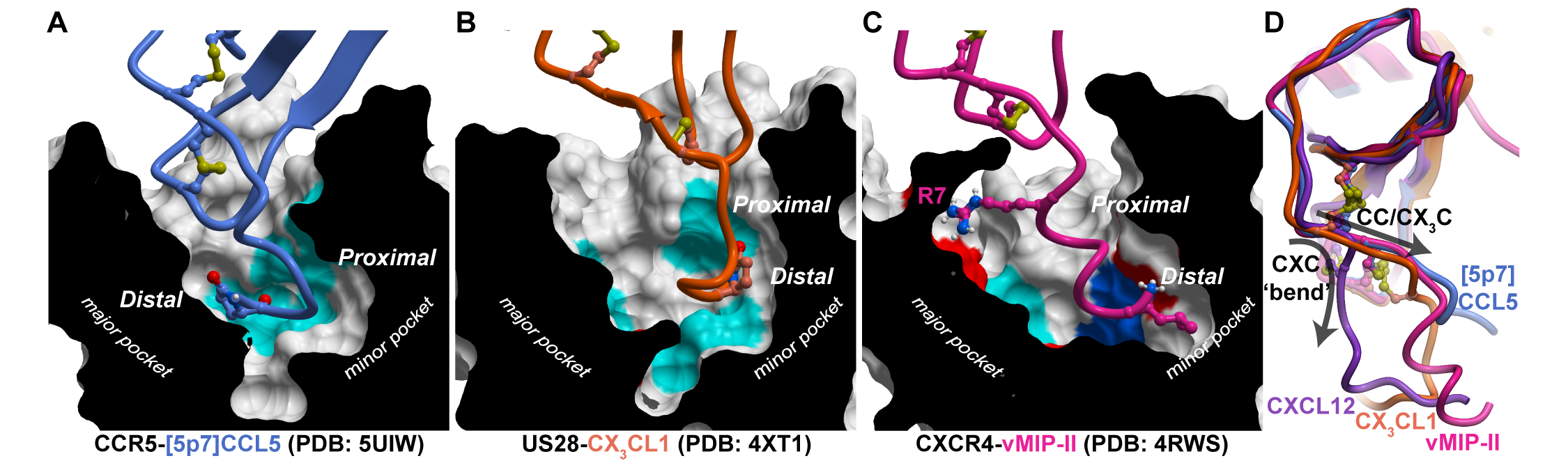

Supplement: S10 Fig — (A–C) Compared to Fig 4G, the proximal N terminus of CC and CX3C chemokines occupy the receptor minor subpocket. vMIP-II is unique among CC chemokines, containing an arginine, conserved in CXC chemokines, allowing it to partially occupy the top of the receptor major subpocket. (D) Overlay of the CC and CX3C chemokines determined in crystal structures, along with CXCL12. CXC chemokines have a pronounced N-terminal bend. (TIF) [file pbio.3000656.s011.tif]

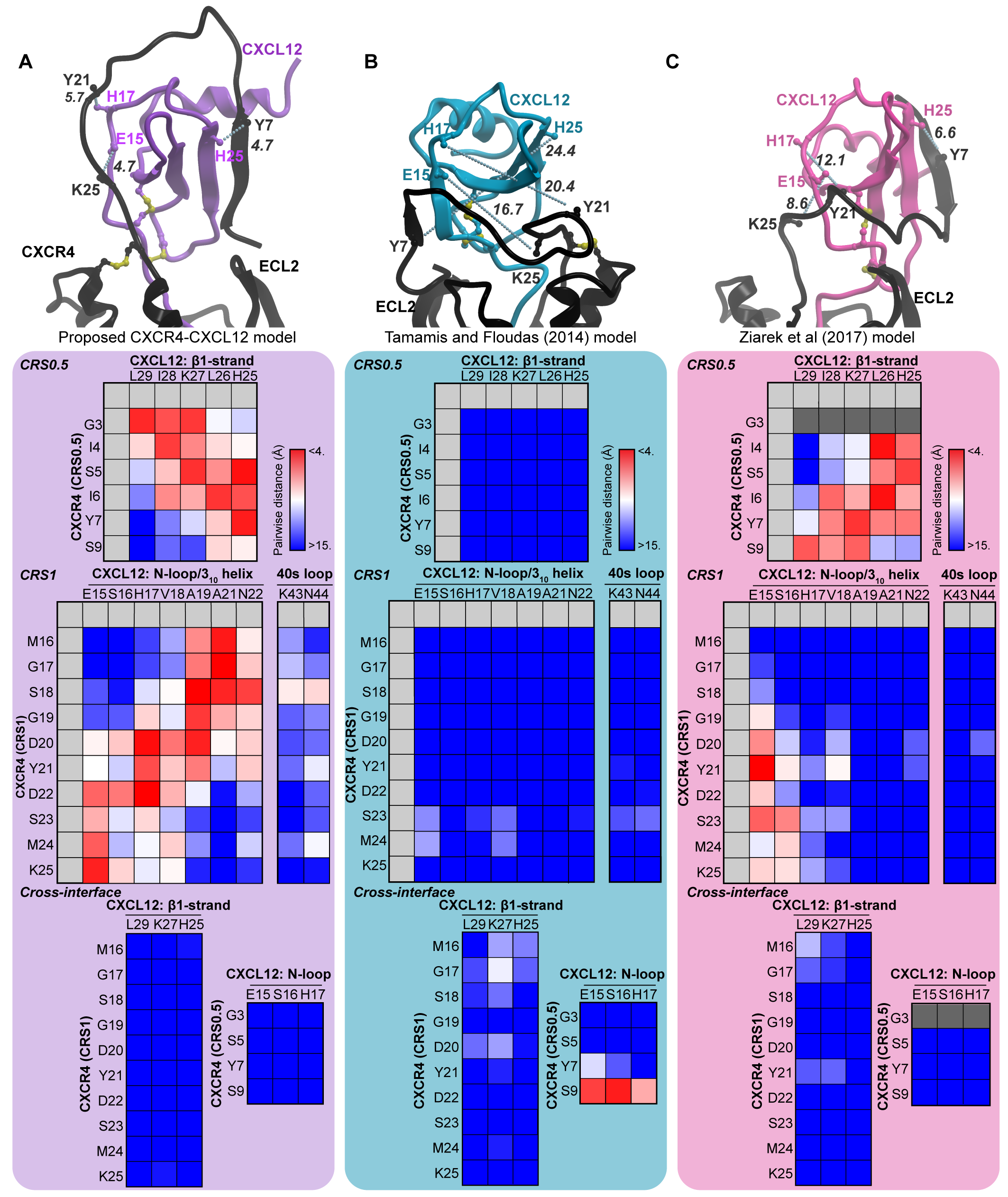

Supplement: S11 Fig — Receptor-chemokine residue Cβ-Cβ (or Cα for Gly) distances were calculated for 3 models of the CXCR4-CXCL12 complex and projected onto a heat map for comparison with experimental crosslinking. (A) The model generated here; (B) the model published by Tamamis and Floudas [51]; (C) the model published by Ziarek and colleagues [25]. We note that the Tamamis and Floudas model was built prior to publication of the CXCR4-vMIP-II crystal structure, and that the Ziarek and colleagues model was informed by NMR of CXCL12 with an isolated N-terminal peptide of CXCR4. In the Ziarek and colleagues model, residue G3 was not modeled (dark gray in the heat map). Cβ-Cβ distances between residue pairs (CXCR4 K25-CXCL12 E15, Y21-H17, and Y7-H25) are shown in blue dotted lines, and their distances are given in Ångstroms. The receptor is shown in black, and CXCL12 is colored differently in each model. The underlying numerical data for each figure panel can be found in S1 Data. NMR, nuclear magnetic resonance. (TIF) [file pbio.3000656.s012.tif]

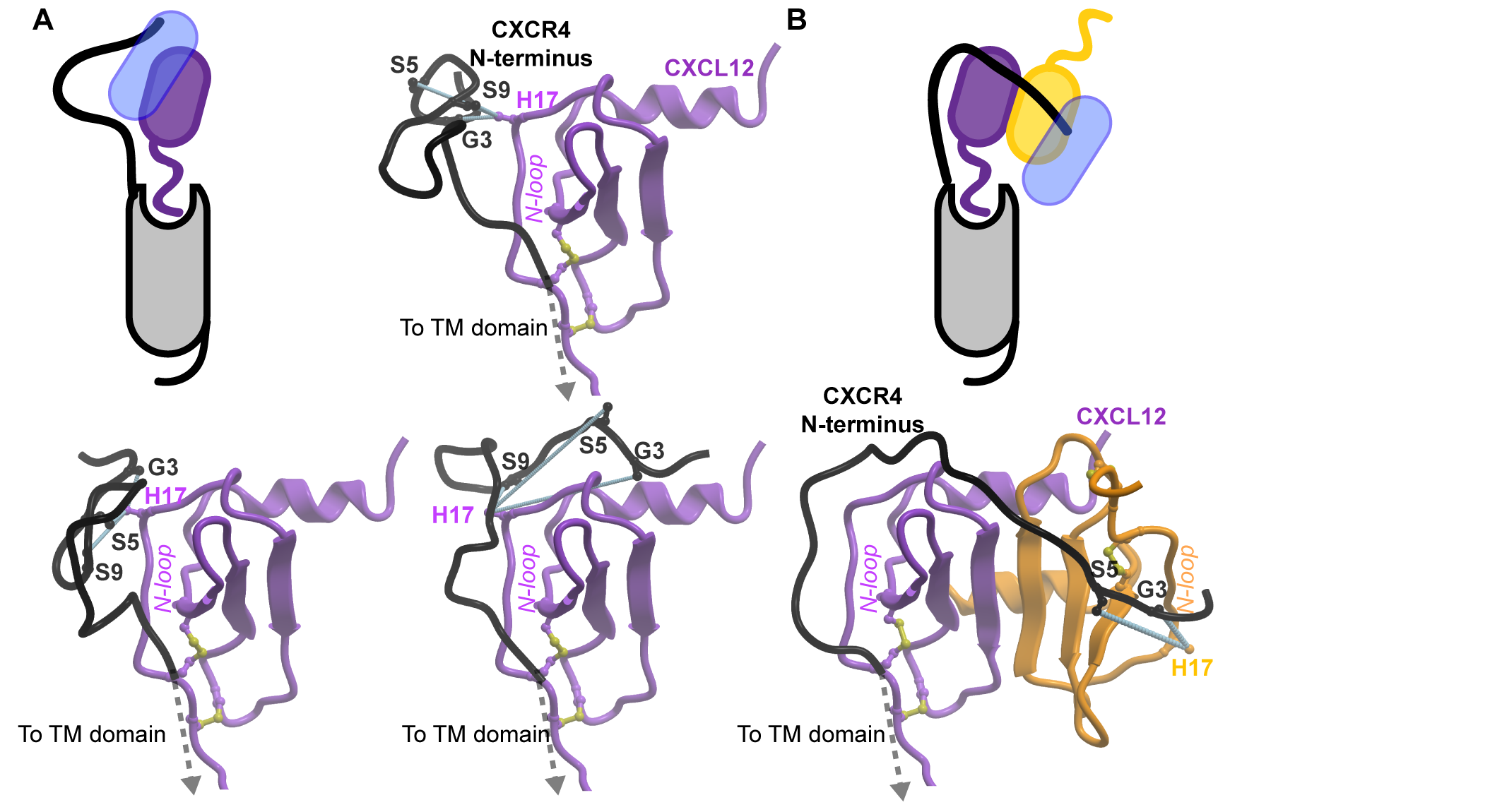

Supplement: S12 Fig — Shown are representative conformations in which the distal N terminus of CXCR4 was found in proximity of the CXCL12 N-loop (A) in the context of the CXCL12 monomer or (B) in the context of the CXCL12 dimer. In panel (B), the distal CXCR4 N terminus potentially interacts with the N-loop of the CXCL12 dimer partner if fully extended. The receptor and CXCL12 are shown in black and purple, respectively. The second monomer in the CXCL12 dimer is shown in orange. Residue proximities reconciled by these alternative models but not by the best-scoring model are shown as light-blue dotted lines. (TIF) [file pbio.3000656.s013.tif]

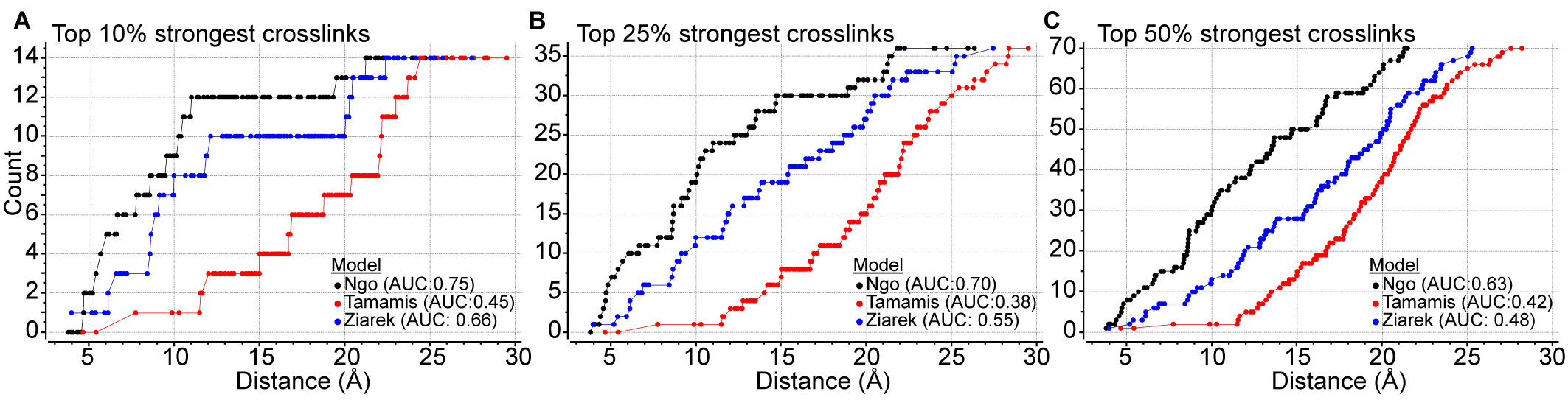

Supplement: S13 Fig — For each model, pairwise residue Cβ-Cβ distances were ranked and receiver operating characteristic curves were generated based on their ability to recognize: (A) the top 10% (14 crosslinks), (B) the top 25% (36), or (C) the top 50% (72) strongest experimentally determined cross-links. Model “Ngo” is our proposed model in this study (S2 Data), and “Tamamis” and “Ziarek” are models published previously [25,51]. The underlying numerical data for each figure panel can be found in S1 Data. (TIF) [file pbio.3000656.s014.tif]

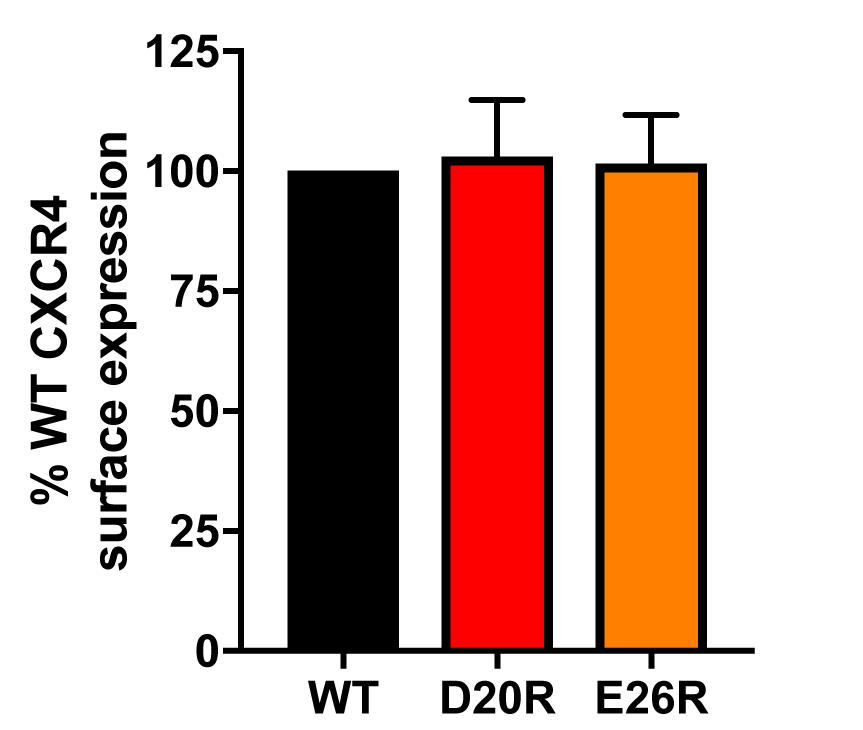

Supplement: S14 Fig — Surface expression was determined by flow cytometry using an allophycocyanin-conjugated anti-CXCR4 antibody and normalized to WT CXCR4. Data represent mean and SEM of n = 4 independent replicates. The underlying numerical data for each figure panel can be found in S1 Data. WT, wild type. (TIF) [file pbio.3000656.s015.tif]

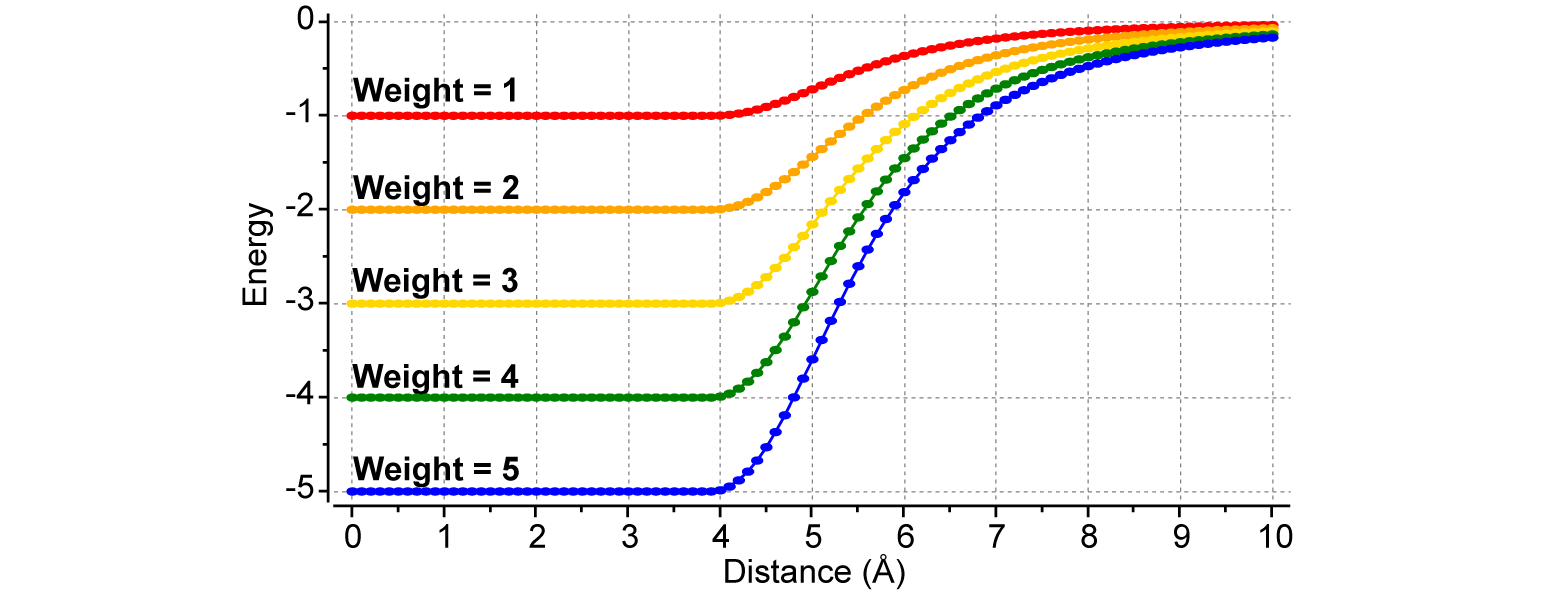

Supplement: S15 Fig — The profile of the restraint penalty features a flat well of varying depth (defined by the restraint weight) at shorter interatomic distances and increases as these distances increase; however, rather than growing indefinitely, the penalty asymptotically approaches 0 as the interatomic distances continue to increase. This allows the sampling procedure to ignore those restraints that cannot be satisfied concurrently with the majority of other restraints and, in this way, resolve conflicts in the experimental data. The underlying numerical data for each figure panel can be found in S1 Data. (TIF) [file pbio.3000656.s016.tif]
