# Supplementary material for: Crosslinking-guided geometry of a complete CXC receptor-chemokine complex and the basis of chemokine subfamily selectivity
Source: PLoS Biol. 2020 Apr 9;18(4):e3000656. doi: 10.1371/journal.pbio.3000656 (PMC7173943; doi:10.1371/journal.pbio.3000656)
Supplement: S1 Table — (DOCX) [file pbio.3000656.s017.docx]

| **Technique** | **Mapping of interface residue roles** | **Full-length receptor?** | **Mutagenesis artifacts** | **References** |
| --- | --- | --- | --- | --- |
| NMR chemical shifts | Single-sided | Challenging | N/A | [26,27,31] |
| NMR NOEs | Pairwise | Challenging | N/A | [24,25,30,49] |
| Transferred cross saturation NMR | Single-sided | Feasible | N/A | [56] |
| Solid state NMR | Single-sided | Feasible | N/A | [57] |
| Site-directed mutagenesis | Single-sided | Feasible | Possible | [22,24,25,33,34,43] |
| Radiolytic footprinting | Single-sided | Feasible | N/A | [34] |
| **Pairwise charge-swap mutagenesis** | **Pairwise** | **Feasible** | **Possible** | **[22]**  **This study** |
| **Disulfide crosslinking** | **Pairwise** | **Feasible** | **Possible** | **This study** |
