# Supplementary material for: Crosslinking-guided geometry of a complete CXC receptor-chemokine complex and the basis of chemokine subfamily selectivity
Source: PLoS Biol. 2020 Apr 9;18(4):e3000656. doi: 10.1371/journal.pbio.3000656 (PMC7173943; doi:10.1371/journal.pbio.3000656)
Supplement: S2 Table — (DOCX) [file pbio.3000656.s018.docx]

| **Residue** | **Domain** | **Flexibility**^a^ | **Mean B-factor (Å^2^)** | **Mean res. SASA (±SD)^b^** |
| --- | --- | --- | --- | --- |
| K1 | N-term | 0.99 | 57.30 | 0.87 ± 0.37 |
| P2 | N-term | 0.98 | 59.46 | 0.82 ± 0.42 |
| V3 | N-term | 0.97 | 55.19 | 0.88 ± 0.47 |
| S4 | N-term | 0.91 | 51.38 | 0.89 ± 0.46 |
| L5 | N-term | 0.91 | 40.65 | 0.86 ± 0.36 |
| S6 | N-term | 0.80 | 32.45 | 0.77 ± 0.36 |
| Y7 | N-term | 0.80 | 32.44 | 0.56 ± 0.28 |
| R8 | N-term | 0.72 | 37.91 | 0.78 ± 0.20 |
| C9 | CxC | 0.30 | 28.66 | 0.30 ± 0.09 |
| P10 | CxC | 0.30 | 28.52 | 0.44 ± 0.12 |
| C11 | CxC | 0.21 | 30.13 | 0.08 ± 0.06 |
| R12 | **N-loop** | 0.75 | 40.35 | 0.74 ± 0.15 |
| F13 | **N-loop** | 0.52 | 28.28 | 0.68 ± 0.09 |
| F14 | **N-loop** | 0.52 | 32.16 | 0.40 ± 0.07 |
| E15 | **N-loop** | 0.32 | 32.58 | 0.36 ± 0.09 |
| S16 | **N-loop** | 0.38 | 34.73 | 0.45 ± 0.10 |
| **H17** | **N-loop** | **0.39** | **39.05** | **0.86 ± 0.05** |
| V18 | **N-loop** | 0.31 | 24.98 | 0.10 ± 0.04 |
| A19 | **N-loop** | 0.24 | 25.29 | 0.47 ± 0.05 |
| R20 | **3_10_ helix** | 0.53 | 34.49 | 0.48 ± 0.10 |
| A21 | **3_10_ helix** | 0.22 | 25.92 | 0.67 ± 0.06 |
| N22 | **3_10_ helix** | 0.34 | 28.12 | 0.29 ± 0.08 |
| V23 | - | 0.32 | 22.49 | 0.08 ± 0.04 |
| K24 | - | 0.42 | 28.00 | 0.51 ± 0.07 |
| H25 | **β1-strand** | 0.43 | 26.26 | 0.42 ± 0.08 |
| **L26** | **β1-strand** | **0.25** | **22.65** | **0.16 ± 0.07** |
| K27 | **β1-strand** | 0.56 | 28.45 | 0.49 ± 0.09 |
| **I28** | **β1-strand** | **0.33** | **22.56** | **0.44 ± 0.06** |
| **L29** | **β1-strand** | **0.34** | **26.63** | **0.22 ± 0.05** |
| N30 | 30s loop | 0.51 | 29.32 | 0.83 ± 0.10 |
| T31 | 30s loop | 0.47 | 29.52 | 0.25 ± 0.14 |
| P32 | 30s loop | 0.69 | 30.98 | 0.71 ± 0.10 |
| N33 | 30s loop | 0.62 | 35.04 | 0.89 ± 0.32 |
| C34 | - | 0.37 | 25.63 | 0.12 ± 0.04 |
| A35 | - | 0.46 | 25.64 | 0.69 ± 0.18 |
| L36 | - | 0.67 | 24.47 | 0.34 ± 0.22 |
| Q37 | β2-strand | 0.61 | 29.17 | 0.16 ± 0.12 |
| I38 | β2-strand | 0.23 | 23.08 | 0.06 ± 0.04 |
| V39 | β2-strand | 0.16 | 22.78 | 0.06 ± 0.02 |
| A40 | β2-strand | 0.15 | 20.41 | 0.00 ± 0.01 |
| R41 | β2-strand | 0.48 | 31.34 | 0.31 ± 0.06 |
| L42 | β2-strand | 0.19 | 23.56 | 0.08 ± 0.03 |
| **K43** | **40s loop** | **0.44** | **30.94** | **0.54 ± 0.06** |
| **N44** | **40s loop** | **0.46** | **35.07** | **0.77 ± 0.12** |
| **N45** | **40s loop** | **0.32** | **32.29** | **0.48 ± 0.08** |
| **N46** | **40s loop** | **0.33** | **33.22** | **0.49 ± 0.06** |
| R47 | β3-strand | 0.57 | 33.84 | 0.61 ± 0.06 |
| Q48 | β3-strand | 0.32 | 29.79 | 0.46 ± 0.06 |
| V49 | β3-strand | 0.22 | 23.06 | 0.11 ± 0.06 |
| C50 | β3-strand | 0.10 | 21.01 | 0.13 ± 0.03 |
| I51 | β3-strand | 0.31 | 24.57 | 0.00 ± 0.00 |
| D52 | - | 0.21 | 27.51 | 0.16 ± 0.06 |
| P53 | - | 0.23 | 28.06 | 0.31 ± 0.10 |
| K54 | - | 0.69 | 32.70 | 0.78 ± 0.07 |
| L55 | - | 0.38 | 27.28 | 0.16 ± 0.05 |
| K56 | αC | 0.65 | 30.14 | 0.80 ± 0.11 |
| W57 | αC | 0.25 | 24.28 | 0.14 ± 0.03 |
| I58 | αC | 0.34 | 25.82 | 0.02 ± 0.02 |
| Q59 | αC | 0.67 | 36.07 | 0.56 ± 0.12 |
| E60 | αC | 0.64 | 37.28 | 0.44 ± 0.09 |
| Y61 | αC | 0.65 | 30.44 | 0.33 ± 0.11 |
| L62 | αC | 0.62 | 26.57 | 0.34 ± 0.13 |
| E63 | αC | 0.74 | 37.08 | 0.53 ± 0.12 |
| K64 | αC | 0.80 | 39.84 | 0.69 ± 0.13 |
| A65 | αC | 0.78 | 33.77 | 0.60 ± 0.23 |
| L66 | αC | 0.87 | 43.14 | 0.74 ± 0.31 |
| N67 | αC | 0.93 | 57.33 | 0.99 ± 0.44 |
| K68 | αC | 0.99 | 66.76 | 0.98 ± 0.41 |

^a^Normalized range from 0 (rigid) to 1 (flexible) – median = 0.38

^b^Normalized range from 0 (buried) to 1 (exposed) – median = 41.88
