# Supplementary material for: Crosslinking-guided geometry of a complete CXC receptor-chemokine complex and the basis of chemokine subfamily selectivity
Source: PLoS Biol. 2020 Apr 9;18(4):e3000656. doi: 10.1371/journal.pbio.3000656 (PMC7173943; doi:10.1371/journal.pbio.3000656)
Supplement: S3 Table — (DOCX) [file pbio.3000656.s019.docx]

| **PDB ID** | **Chain** | **Resolution (Å)** | **Length** | **Cluster** |
| --- | --- | --- | --- | --- |
| 1A15 | A | 2.2 | 67 | 1 |
| **1QG7** | **A*** | **2** | **62** | **1** |
| 1A15 | B | 2.2 | 57 | 2 |
| 1QG7 | B | 2 | 66 | 2 |
| 2NWG | B | 2.07 | 64 | 2 |
| 2J7Z | A | 1.95 | 68 | 2 |
| 2NWG | A | 2.07 | 68 | 2 |
| **4UAI** | **A*** | **1.9** | **68** | **2** |
| 3GV3 | A | 1.6 | 63 | 2 |
| 2J7Z | B | 1.95 | 68 | 2 |
| 4UAI | B | 1.9 | 67 | 2 |
| 4LMQ | D | 2.77 | 55 | 3 |
| 3HP3 | C | 2.2 | 64 | 3 |
| 3HP3 | H | 2.2 | 64 | 3 |
| **3HP3** | **F*** | **2.2** | **65** | **3** |
| 3HP3 | I | 2.2 | 65 | 3 |
| 3HP3 | J | 2.2 | 63 | 3 |
| 3HP3 | D | 2.2 | 64 | 3 |
| 3HP3 | B | 2.2 | 62 | 3 |
| 3HP3 | E | 2.2 | 61 | 3 |
| 3HP3 | G | 2.2 | 65 | 3 |
| **3HP3** | **A*** | **2.2** | **64** | **4** |
| **4LMQ** | **F*** | **2.77** | **58** | **5** |

Each cluster is highlighted with a different color. *****Indicates the CXCL12 crystal structure chain of each cluster used as alternative backbone conformations in S8 Fig.
