# Supplementary material for: Crosslinking-guided geometry of a complete CXC receptor-chemokine complex and the basis of chemokine subfamily selectivity
Source: PLoS Biol. 2020 Apr 9;18(4):e3000656. doi: 10.1371/journal.pbio.3000656 (PMC7173943; doi:10.1371/journal.pbio.3000656)
Supplement: S4 Table — (DOCX) [file pbio.3000656.s020.docx]

| **CXCR4 mutant** | **Chemokine domain** | **[P2G]CXCL12 mutant** | **Crosslinking efficiency % (flow cytometry)^a^** | **Crosslinking efficiency % (Western blot)** | **Fraction receptor crosslinked, % (Western blot)** | **Distance restraint weight^b^** |
| --- | --- | --- | --- | --- | --- | --- |
| G3C | **β1-strand** | L29C | 107.2 ± 9.3 | 68.3 ± 4.7 | 45.5 ± 0.4 | 3.18 |
| G3C | **β1-strand** | I28C | 19.8 ± 2.7 | ND | ND | - |
| G3C | **β1-strand** | K27C | 135.5 ± 7.2 | 104.9 ± 10.8 | 49.1 ± 1.9 | 5.00 |
| G3C | **β1-strand** | L26C | 22.0 ± 2.4 | ND | ND | - |
| G3C | **β1-strand** | H25C | 81.7 ± 5.9 | ND | ND | 1.54 |
| I4C | **β1-strand** | L29C | 42.7 ± 6.6 | ND | ND | - |
| I4C | **β1-strand** | I28C | 20.7 ± 3.1 | ND | ND | - |
| I4C | **β1-strand** | K27C | 39.4 ± 2.5 | ND | ND | - |
| I4C | **β1-strand** | L26C | 22.7 ± 6.3 | ND | ND | - |
| I4C | **β1-strand** | H25C | 69.9 ± 6.0 | ND | ND | 0.78 |
| S5C | **β1-strand** | L29C | 33.6 ± 2.7 | N/A | N/A | - |
| S5C | **β1-strand** | I28C | 15.9 ± 2.6 | ND | ND | - |
| S5C | **β1-strand** | K27C | 66.9 ± 5.7 | 56.5 ± 2.4 | 49.0 ± 2.1 | 0.58 |
| S5C | **β1-strand** | L26C | 16.5 ± 4.1 | ND | ND | - |
| S5C | **β1-strand** | H25C | 74.2 ± 10.4 | 69.7 ± 4.9 | 43.7 ± 6.9 | 1.06 |
| I6C | **β1-strand** | L29C | 24.5 ± 2.2 | ND | ND | - |
| I6C | **β1-strand** | I28C | 14.0 ± 2.2 | ND | ND | - |
| I6C | **β1-strand** | K27C | 36.5 ± 7.6 | ND | ND | - |
| I6C | **β1-strand** | L26C | 16.7 ± 1.9 | ND | ND | - |
| I6C | **β1-strand** | H25C | 36.1 ± 5.8 | ND | ND | - |
| Y7C | **β1-strand** | L29C | 27.2 ± 2.2 | ND | ND | - |
| Y7C | **β1-strand** | I28C | 16.8 ± 2.8 | ND | ND | - |
| Y7C | **β1-strand** | K27C | 62.5 ± 5.3 | 16.7 ± 2.0 | 26.9 ± 2.2 | - |
| Y7C | **β1-strand** | L26C | 19.2 ± 2.9 | ND | ND | - |
| Y7C | **β1-strand** | H25C | 88.0 ± 5.3 | 98.2 ± 13.0 | 41.1 ± 9.0 | 1.94 |
| S9C | **β1-strand** | L29C | 47.9 ± 6.9 | 13.2 ± 2.4 | 14.1 ± 1.0 | - |
| S9C | **β1-strand** | I28C | 18.0 ± 4.2 | ND | ND | - |
| S9C | **β1-strand** | K27C | 64.1 ± 11.8 | ND | ND | - |
| S9C | **β1-strand** | L26C | 15.2 ± 3.2 | ND | ND | - |
| S9C | **β1-strand** | H25C | 81.2 ± 8.5 | 90.5 ± 2.7 | 43.5 ± 10.4 | 1.51 |
| M16C | **N-loop** | E15C | 53.1 ± 10.7 | ND | ND | - |
| M16C | **N-loop** | S16C | 50.4 ± 1.8 | ND | ND | - |
| M16C | **N-loop** | H17C | 61.5 ± 2.5 | ND | ND | - |
| M16C | **N-loop** | V18C | 56.7 ± 3.8 | ND | ND | - |
| M16C | **3_10_ helix** | A19C | 51.9 ± 9.1 | ND | ND | - |
| M16C | **3_10_ helix** | A21C | 72.7 ± 7.2 | 95.1 ± 12.2 | 60.7 ± 3.8 | 0.96 |
| M16C | **3_10_ helix** | N22C | 63.4 ± 3.1 | ND | ND | - |
| M16C | **40s loop** | K43C | 22.6 ± 0.6 | ND | ND | - |
| M16C | **40s loop** | N44C | 47.0 ± 7.4 | ND | ND | - |
| G17C | **N-loop** | E15C | 56.6 ± 4.8 | ND | ND | - |
| G17C | **N-loop** | S16C | 48.0 ± 4.7 | ND | ND | - |
| G17C | **N-loop** | H17C | 65.6 ± 7.8 | ND | ND | 0.50 |
| G17C | **N-loop** | V18C | 66.2 ± 4.0 | ND | ND | 0.54 |
| G17C | **3_10_ helix** | A19C | 48.9 ± 8.2 | ND | ND | - |
| G17C | **3_10_ helix** | A21C | 64.6 ± 6.7 | 92.5 ± 14.0 | 66.4 ± 6.3 | - |
| G17C | **3_10_ helix** | N22C | 57.4 ± 2.4 | ND | ND | - |
| G17C | **40s loop** | K43C | 21.2 ± 0.5 | ND | ND | - |
| G17C | **40s loop** | N44C | 50.3 ± 6.5 | ND | ND | - |
| S18C | **N-loop** | E15C | 52.9 ± 14.2 | ND | ND | - |
| S18C | **N-loop** | S16C | 79.6 ± 14.4 | ND | ND | 1.40 |
| S18C | **N-loop** | H17C | 83.1 ± 4.8 | ND | ND | 1.63 |
| S18C | **N-loop** | V18C | 39.3 ± 6.1 | ND | ND | - |
| S18C | **3_10_ helix** | A19C | 37.3 ± 3.6 | ND | ND | - |
| S18C | **3_10_ helix** | A21C | 67.9 ± 9.0 | ND | ND | 0.65 |
| S18C | **3_10_ helix** | N22C | 97.9 ± 3.1 | 162.6 ± 16.0 | 58.5 ± 4.3 | 2.58 |
| S18C | **40s loop** | K43C | 34.9 ± 3.9 | ND | ND | - |
| S18C | **40s loop** | N44C | 73.4 ± 5.1 | 70.6 ± 11.7 | 51.1 ± 17.3 | 1.00 |
| G19C | **N-loop** | E15C | 41.8 ± 1.8 | ND | ND | - |
| G19C | **N-loop** | S16C | 66.7 ± 8.4 | ND | ND | 0.57 |
| G19C | **N-loop** | H17C | 79.0 ± 10.2 | ND | ND | 1.36 |
| G19C | **N-loop** | V18C | 71.1 ± 3.3 | ND | ND | 0.85 |
| G19C | **3_10_ helix** | A19C | 27.2 ± 2.2 | ND | ND | - |
| G19C | **3_10_ helix** | A21C | 31.9 ± 2.2 | ND | ND | - |
| G19C | **3_10_ helix** | N22C | 58.8 ± 7.0 | ND | ND | - |
| G19C | **40s loop** | K43C | 22.0 ± 1.9 | ND | ND | - |
| G19C | **40s loop** | N44C | 57.3 ± 13.4 | ND | ND | - |
| D20C | **N-loop** | E15C | 46.5 ± 4.5 | ND | ND | - |
| D20C | **N-loop** | S16C | 52.7 ± 18.4 | ND | ND | - |
| D20C | **N-loop** | H17C | 44.5 ± 8.1 | ND | ND | - |
| D20C | **N-loop** | V18C | 52.0 ± 0.4 | ND | ND | - |
| D20C | **3_10_ helix** | A19C | 37.0 ± 0.8 | ND | ND | - |
| D20C | **3_10_ helix** | A21C | 28.4 ± 3.6 | ND | ND | - |
| D20C | **3_10_ helix** | N22C | 52.0 ± 2.7 | ND | ND | - |
| D20C | **40s loop** | K43C | 24.9 ± 2.9 | ND | ND | - |
| D20C | **40s loop** | N44C | 36.5 ± 9.5 | ND | ND | - |
| Y21C | **N-loop** | E15C | 83.6 ± 18.9 | ND | ND | 1.66 |
| Y21C | **N-loop** | S16C | 111.4 ± 15.4 | ND | ND | 3.45 |
| Y21C | **N-loop** | H17C | 86.0 ± 9.7 | ND | ND | 1.82 |
| Y21C | **N-loop** | V18C | 41.9 ± 4.6 | ND | ND | - |
| Y21C | **3_10_ helix** | A19C | 34.8 ± 2.1 | ND | ND | - |
| Y21C | **3_10_ helix** | A21C | 41.2 ± 4.3 | ND | ND | - |
| Y21C | **3_10_ helix** | N22C | 55.8 ± 4.1 | 30.7 ± 5.1 | 40.3 ± 3.9 | - |
| Y21C | **40s loop** | K43C | 41.2 ± 4.2 | ND | ND | - |
| Y21C | **40s loop** | N44C | 34.8 ± 1.8 | 24.9 ± 7.5 | 46.4 ± 5.9 | - |
| D22C | **N-loop** | E15C | 33.8 ± 4.7 | ND | ND | - |
| D22C | **N-loop** | S16C | 90.2 ± 18.6 | ND | ND | 2.09 |
| D22C | **N-loop** | H17C | 61.1 ± 6.7 | ND | ND | - |
| D22C | **N-loop** | V18C | 35.9 ± 6.2 | ND | ND | - |
| D22C | **3_10_ helix** | A19C | 28.1 ± 0.8 | ND | ND | - |
| D22C | **3_10_ helix** | A21C | 30.9 ± 4.4 | ND | ND | - |
| D22C | **3_10_ helix** | N22C | 56.8 ± 1.8 | ND | ND | - |
| D22C | **40s loop** | K43C | 37.8 ± 4.4 | ND | ND | - |
| D22C | **40s loop** | N44C | 28.8 ± 1.5 | ND | ND | - |
| S23C | **N-loop** | E15C | 84.9 ± 19.1 | ND | ND | 1.74 |
| S23C | **N-loop** | S16C | 82.4 ± 7.1 | ND | ND | 1.58 |
| S23C | **N-loop** | H17C | 67.3 ± 9.7 | ND | ND | 0.61 |
| S23C | **N-loop** | V18C | 44.4 ± 6.6 | ND | ND | - |
| S23C | **3_10_ helix** | A19C | 36.8 ± 2.9 | ND | ND | - |
| S23C | **3_10_ helix** | A21C | 34.1 ± 2.6 | ND | ND | - |
| S23C | **3_10_ helix** | N22C | 63.5 ± 2.3 | ND | ND | - |
| S23C | **40s loop** | K43C | 52.1 ± 4.7 | ND | ND | - |
| S23C | **40s loop** | N44C | 44.8 ± 3.0 | ND | ND | - |
| M24C | **N-loop** | E15C | 96.7 ± 22.0 | 45.6 ± 9.7 | 46.5 ± 3.0 | 2.50 |
| M24C | **N-loop** | S16C | 105.7 ± 16.7 | 79.2 ± 17.1 | 61.1 ± 4.5 | 3.08 |
| M24C | **N-loop** | H17C | 72.6 ± 7.1 | ND | ND | 0.95 |
| M24C | **N-loop** | V18C | 32.4 ± 4.3 | ND | ND | - |
| M24C | **3_10_ helix** | A19C | 36.1 ± 3.1 | ND | ND | - |
| M24C | **3_10_ helix** | A21C | 34.8 ± 2.0 | ND | ND | - |
| M24C | **3_10_ helix** | N22C | 43.0 ± 0.9 | ND | ND | - |
| M24C | **40s loop** | K43C | 48.4 ± 4.6 | ND | ND | - |
| M24C | **40s loop** | N44C | 38.3 ± 3.2 | ND | ND | - |
| K25C | **N-loop** | E15C | 121.4 ± 22.2 | 116.7 ± 11.7 | 50.7 ± 9.0 | 4.09 |
| K25C | **N-loop** | S16C | 103.9 ± 15.5 | 128.0 ± 16.5 | 72.1 ± 7.6 | 2.96 |
| K25C | **N-loop** | H17C | 50.9 ± 5.4 | ND | ND | - |
| K25C | **N-loop** | V18C | 28.9 ± 3.8 | ND | ND | - |
| K25C | **3_10_ helix** | A19C | 18.6 ± 0.9 | ND | ND | - |
| K25C | **3_10_ helix** | A21C | 26.4 ± 2.5 | 4.1 ± 0.7 | 20.6 ± 3.7 | - |
| K25C | **3_10_ helix** | N22C | 27.1 ± 1.2 | ND | ND | - |
| K25C | **40s loop** | K43C | 48.6 ± 3.1 | ND | ND | - |
| K25C | **40s loop** | N44C | 23.2 ± 1.4 | ND | ND | - |
| G3C | **N-loop** | E15C | 31.0 ± 3.1 | ND | ND | - |
| G3C | **N-loop** | S16C | 39.6 ± 7.0 | ND | ND | - |
| G3C | **N-loop** | H17C | 69.3 ± 12.8 | ND | ND | 0.74 |
| S5C | **N-loop** | E15C | 30.2 ± 5.3 | ND | ND | - |
| S5C | **3_10_ helix** | S16C | 45.3 ± 7.0 | ND | ND | - |
| S5C | **3_10_ helix** | H17C | 67.7 ± 11.8 | ND | ND | 0.64 |
| Y7C | **3_10_ helix** | E15C | 38.6 ± 4.8 | ND | ND | - |
| Y7C | **40s loop** | S16C | 56.0 ± 5.7 | ND | ND | - |
| Y7C | **40s loop** | H17C | 92.5 ± 13.0 | ND | ND | 2.23 |
| S9C | **N-loop** | E15C | 47.3 ± 4.9 | ND | ND | - |
| S9C | **N-loop** | S16C | 61.9 ± 8.1 | ND | ND | - |
| S9C | **N-loop** | H17C | 107.7 ± 16.3 | 29.8 ± 9.1 | 39.0 ± 9.7 | 3.21 |
| M16C | **β1-strand** | L29C | 26.5 ± 5.9 | ND | ND | - |
| M16C | **β1-strand** | K27C | 42.5 ± 13.5 | ND | ND | - |
| M16C | **β1-strand** | H25C | 68.0 ± 4.7 | ND | ND | 0.65 |
| G17C | **β1-strand** | L29C | 24.4 ± 7.2 | ND | ND | - |
| G17C | **β1-strand** | K27C | 47.5 ± 15.3 | ND | ND | - |
| G17C | **β1-strand** | H25C | 52.7 ± 16.4 | ND | ND | - |
| S18C | **β1-strand** | L29C | 32.8 ± 1.8 | ND | ND | - |
| S18C | **β1-strand** | K27C | 59.9 ± 10.5 | ND | ND | - |
| S18C | **β1-strand** | H25C | 52.0 ± 19.3 | ND | ND | - |
| G19C | **β1-strand** | L29C | 37.5 ± 2.0 | ND | ND | - |
| G19C | **β1-strand** | K27C | 76.0 ± 5.9 | 18.4 ± 5.9 | 39.5 ± 14.1 | 1.17 |
| G19C | **β1-strand** | H25C | 47.1 ± 17.7 | ND | ND | - |
| D20C | **β1-strand** | L29C | 26.2 ± 8.1 | ND | ND | - |
| D20C | **β1-strand** | K27C | 41.0 ± 15.8 | ND | ND | - |
| D20C | **β1-strand** | H25C | 62.5 ± 4.8 | ND | ND | - |
| Y21C | **β1-strand** | L29C | 45.1 ± 4.0 | ND | ND | - |
| Y21C | **β1-strand** | K27C | 56.5 ± 11.3 | ND | ND | - |
| Y21C | **β1-strand** | H25C | 81.2 ± 12.6 | ND | ND | 1.50 |
| D22C | **β1-strand** | L29C | 32.8 ± 2.1 | ND | ND | - |
| D22C | **β1-strand** | K27C | 58.7 ± 4.6 | ND | ND | - |
| D22C | **β1-strand** | H25C | 62.7 ± 6.7 | ND | ND | - |
| S23C | **β1-strand** | L29C | 41.0 ± 2.6 | ND | ND | - |
| S23C | **β1-strand** | K27C | 52.2 ± 4.6 | ND | ND | - |
| S23C | **β1-strand** | H25C | 69.0 ± 5.4 | ND | ND | 0.72 |
| M24C | **β1-strand** | L29C | 37.6 ± 3.3 | ND | ND | - |
| M24C | **β1-strand** | K27C | 42.2 ± 4.1 | ND | ND | - |
| M24C | **β1-strand** | H25C | 53.2 ± 2.1 | ND | ND | - |
| K25C | **β1-strand** | L29C | 36.8 ± 5.5 | ND | ND | - |
| K25C | **β1-strand** | K27C | 38.4 ± 3.6 | ND | ND | - |
| K25C | **β1-strand** | H25C | 25.6 ± 1.9 | ND | ND | - |

^a^ Geometric mean fluorescence intensities (GMFI) for anti-HA ([P2G]CXCL12) or anti-Flag (CXCR4) staining of complex-expressing *Sf9* cells were normalized to CXCR4(D187C)-vMIP-II(W5C)[6] in the same experiment. Crosslinking efficiencies by flow cytometry are colored by a gradient of blue to red from low to high efficiency.

^b^ For receptor-chemokine pairs that displayed >65% crosslinking efficiencies by flow cytometry, anti-HA ([P2G]CXCL12) GMFI were normalized and transformed to distance restraint weights that ranged from 0.5 to 5.

ND = not determined

N/A = not applicable
