# Supplementary material for: Crosslinking-guided geometry of a complete CXC receptor-chemokine complex and the basis of chemokine subfamily selectivity
Source: PLoS Biol. 2020 Apr 9;18(4):e3000656. doi: 10.1371/journal.pbio.3000656 (PMC7173943; doi:10.1371/journal.pbio.3000656)
Supplement: Attachment — Submitted filename: Ngo_PLOS_R2R.pdf [file pbio.3000656.s023.pdf]

**Reviewer #1 critiques and responses**

1) I have a concern about the application of flow cytometry to detect the occurrence of crosslinking. Under the assumption that [P2G]CXCL12 dissociates from the receptor when not covalently bound, authors quantify the fluorescent signal given by the cells treated with an antibody targeted to the ligand. This is a delicate step, which in my opinion deserves a better explanation. It is unclear when the ligand that is not covalently bound should dissociate. According to the experimental section, there is no washing step. How do the authors know that this antagonist dissociates so fast? Are there data for the koff of this ligand?

**Response:** This is an important point. Several lines of evidence support rapid dissociation of CXCL12 and especially [P2G]CXCL12 from CXCR4 in several different systems. First, for CXCR4 reconstituted in purified viral particles, the dissociative half-life of WT CXCL12 is estimated at ~1.4 min by surface plasmon resonance [Vega et al., J Leukoc Biol 2011]. Also, in our own experiments in insect cells, we have previously demonstrated that CXCL12 is undetectable on the surface in the absence of crosslinking [Kufareva et al., Methods Enzymol 2016]. In the present study, numerous combinations of [P2G]CXCL12 Cys mutants with CXCR4 Cys mutants led to undetectable chemokine fluorescence signal (Fig 2 in blue). We are aware of one study with somewhat contradictory findings, where CXCL12 dissociation from CXCR4-expressing cells was at least slow enough to enable detection of compound binding by competition [Schoofs et al., J Vis Exp 2018]; however, because in that study, mammalian (Jurkat) cells were employed, the presence of endogenous G proteins in stoichiometric excess likely led to stabilization of receptor-chemokine complexes at the cell surface. In fact, it is known that being coupled to G proteins dramatically improves receptor binding affinity to agonists [Nijmeijer et al., J Biol Chem 2010, Fig 7A] and reduces agonist dissociation rates [DeVree et al., Nature 2016]. In other words, the experimental setup by [Schoofs et al., J Vis Exp 2018] is not directly comparable to ours, and in our setup, the uncrosslinked chemokine dissociation from CXCR4 proved to be very fast.

The fast dissociation rate of CXCL12 and [P2G]CXCL12 from CXCR4-expressing insect cells is in stark contrast with a homolog of CXCR4, the atypical chemokine receptor ACKR3. With ACKR3-expressing insect cells, there is a clear observable signal for most of the mutants, despite the absence of crosslinking (S3 Fig). We have recently demonstrated that CXCL12 has an extremely dissociative half-life at ACKR3 (102 ± 18 min) [Gustavsson et al., Sci Signal 2019], i.e. ~70 (or more) times longer than at CXCR4. This is consistent with the atypical nature of ACKR3 and its proposed biological role as CXCL12 scavenger [Luker et al., Oncogene 2012]; and this explains why the chemokine is detectable on the surface of ACKR3-expressing cells even in the absence of crosslinking.

2) Also, how do the authors take into account the likely different expression level of the different Cys-receptor mutants and Cys-chemokines in the quantification of the flow cytometry data? In this frame, giving crosslinking efficiency with an accuracy to the second decimal place (Supplementary Table 4) may not be appropriate.

**Response:** Firstly, we would like to clarify that even though there is no method for quantifying the expression of the Cys-chemokines in Sf9 cells per se, we strived to ensure that they are in stoichiometric excess with respect to the Cys-receptors. The excess chemokine that is expressed but not crosslinked to the receptor is secreted into the medium and rapidly degraded.

The expression levels of the Cys-receptors pose a more challenging question. We have probed these expression levels by anti-FLAG, and discovered that these expression levels are not inherent to the receptor mutants themselves, but instead are highly variable and partially dependent on the co-expressed chemokine, which is expected given that favorably crosslinked complexes are more stable and better retained on the cell surface. In these conditions, normalizing the cell fluorescent signal by the receptor expression level in the same sample largely cancels the informative variation between receptor-chemokine pairs, and reduces such variation to noise. Because of this, we decided to use the anti-HA signal as is. While variations in receptor expression levels may

partially contribute to this signal, higher receptor expression is indicative of more favorable, stable complexes; therefore, such variation provides useful information for our modeling effort.

We agree that reporting crosslinking efficiency to the second decimal place is not appropriate, and corrected it in S4 Table in the revision.

3) Authors select a smaller subset of ligand-receptor combinations and validate flow cytometry data using western blot to detect the occurrence of crosslinking.

In the modeling experiments, authors apply constraints for pairs displaying >65% crosslinking efficiency (65% in cytometry or WB?).

**Response:** We apologize for the confusion in the initial manuscript. In fact, our distance restraints weights were based only on flow cytometry, and included the receptor-chemokine pairs which demonstrated >65% crosslinking efficiency in the flow cytometry experiments. No receptor-chemokine pairs meeting this criterion were excluded. Western blot data was not used to determine the weights of the distance restraints. This decision was motivated by the desire to (i) collect a relatively large and uniform set of restraints via a single method (flow cytometry), and (ii) to demonstrate that this less labor-intensive approach allows acquisition of sufficiently high quality data to inform modeling. We have now corrected the corresponding sentences in the manuscript. Additionally, to clarify the process of crosslink quantification and distance restraint determination, we have expanded S4 Table to include (a) the crosslinking efficiency determined by flow cytometry (based on anti-HA geometric mean fluorescence intensity), and (b) the weights of the distance restraints, derived from the crosslinking efficiency by flow cytometry.

Authors state, page 10 lines 207-208, that WB data were in line with flow cytometry data. I have trouble agreeing with this statement. In Figure 3, the pair G3C-receptor/L29-ligand would be a hit according to flow cytometry, but not according to WB analysis. There are striking differences also in the pair M24/E15, as well as S9/H17. Importantly, these outliers are "red spots" in the flow cytometry data. Supplementary Fig. 4 is somewhat misleading, because the worst outlier S18/N22 is in fact not problematic, as it appears as a clear hit using both methods.

**Response:** In the process of manuscript revision, and based on reviewers' comments, we realized that the approach that we used to quantify receptor-chemokine crosslinking efficiency introduced a lot of confusion. As a reminder, in the original manuscript, the equation used for quantification had two factors: (i) the intensity of anti-HA (chemokine) bands on the blot, and (ii) the ratio of the intensity of the higher molecular weight anti-Flag band (crosslinked receptor) to total anti-Flag intensity. The first factor (anti-HA staining) is relatable to the anti-HA fluorescence observed on whole cells in flow cytometry. However, the second factor (higher-to-total receptor band intensity ratio) is conceptually orthogonal, as it reports on an unrelated property of the complexes (namely, fraction of receptor crosslinked) that is *only* measurable in Western blotting and not in flow cytometry. Our attempt, in the original manuscript, to factor that second measurement into Western blot quantification made the data hard to interpret and relate between the two methods.

In the revision, we have modified our approach to Western blot analysis and have kept these two aspects separate (amount of chemokine and crosslinked receptor and ratio of crosslinked receptor to total extracted receptor). Because in flow cytometry, crosslinking efficiency was determined by the detection of the chemokine HA tag with an anti-HA FITC-conjugated antibody, the most appropriate direct comparison in the Western blot is the chemokine HA signal as detected by the anti-HA primary and IRDye® 800CW-conjugated secondary antibodies (green bands in Fig 3A, included below). As we can see from the scatter plot in S4A Fig, overall, the magnitude between the two methods reporting the chemokine HA tag is consistent and correlated. Separately,

we added an additional panel (Fig 3C) showing the ratio of crosslinked receptor to total receptor relative to the positive control CXCR4(D187C)-vMIP-II(W5C). This was determined by calculating the fluorescence from the upper receptor FLAG band over the fluorescence from the upper and lower receptor FLAG bands; detailed quantification is addressed in Reviewer 3 Minor comment #5. We thought it was important to include this information. We have now substantially modified the figures (Fig. 3B, 3C and S4 Fig) and manuscript text to reflect these changes, and provided raw numbers (including the crosslinking efficiency determined by Western blot, a.k.a. relative anti-HA blot intensity, and including fraction receptor crosslinked) in S4 Table.

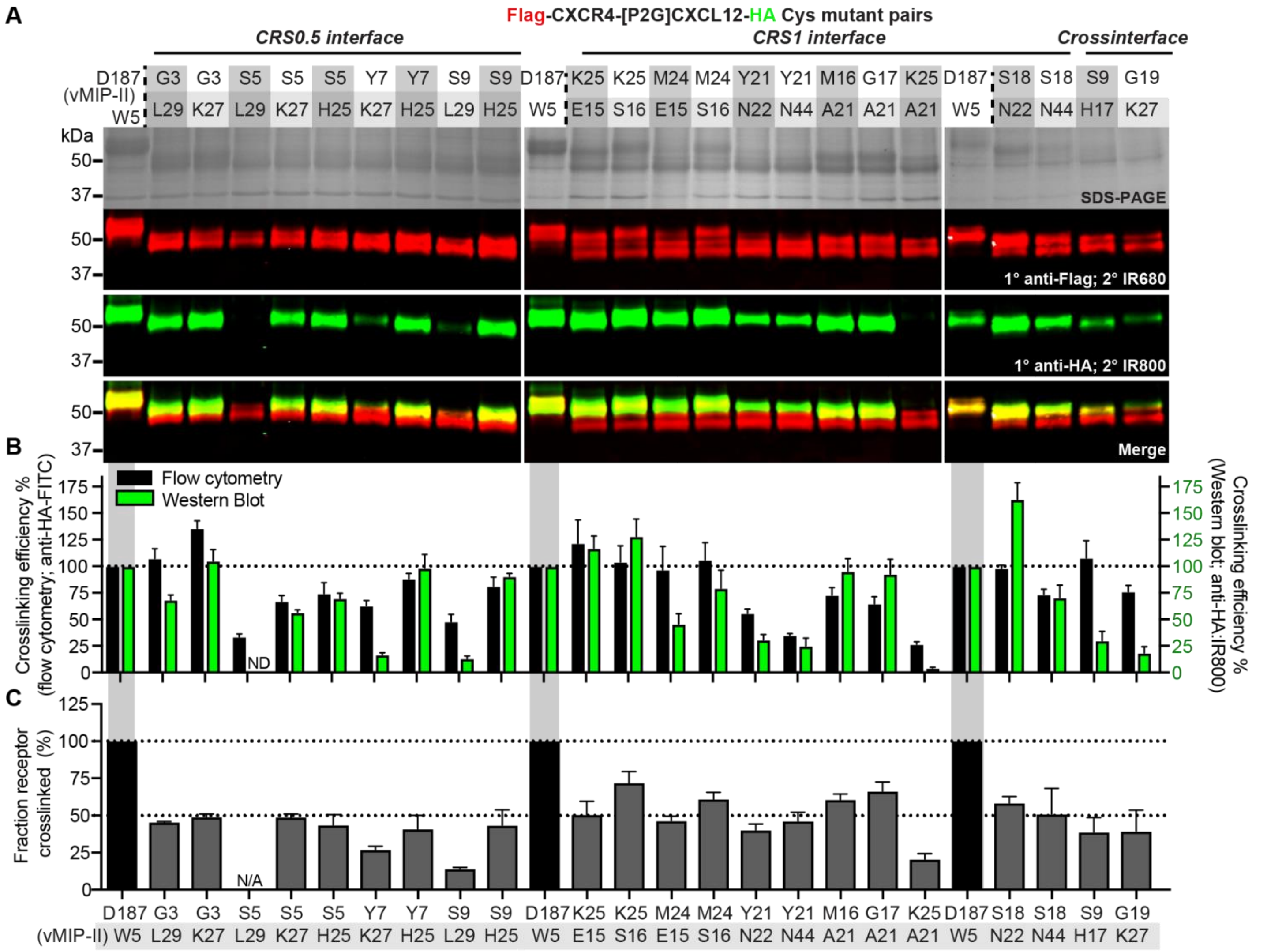


Without the “fraction-receptor-crosslinked” factor, the relationship between flow cytometry and pull-down/Western blot quantification of the receptor-chemokine pairs has somewhat improved (S4A Fig). In particular, for the pairs highlighted by the Reviewer, it is as follows:

- G3/L29: 107.2 ± 9.3 (FC) and 68.3 ± 4.7 (PD/WB) (this pair is in agreement)
- M24/E15: 96.7 ± 22.0 (FC) and 45.6 ± 9.7 (PD/WB) (this pair is only mildly "discordant")
- S9/H17: 107.7 ± 16.3 (FC) and 29.8 ± 9.1 (PD/WB) (this pair remains strongly "discordant")

Other mildly discordant pairs include:

- Y7/K27: 62.5 ± 5.3(FC) and 16.7 ± 2.0 (PD/WB)
- S9/L29: 47.9 ± 6.9 (FC) and 13.2 ± 2.4 (PD/WB)
- G19/K27: 76.0 ± 5.9 (FC) and 18.4 ± 5.9 (PD/WB)

These discrepancies likely indicate that the pull-down/Western blotting approach is less tolerant towards sub-optimal geometry complexes, as compared to flow cytometry. Importantly, modeling/docking is the critical step that helps resolve these ambiguities - see our response to the next comment.

It is not clear how the authors treated these discordant data when refining their model. For instance, was S9/H17 treated as a hit or not? According to the WB data, this pair is not a hit, so that data in figure 2f do not contradict the model.

**Response:** For model refinement, all receptor-chemokine pairs that displayed >65% crosslinking efficiency in flow cytometry were converted into weighted local distance restraints, regardless of whether or not they were discordant with the quantitation by pull-down and Western experiments.

Interestingly, although our modeling process strictly relied on the flow-cytometry-derived proximities; in those cases where Western blotting quantitation disagreed with flow cytometry, the 3D model was largely in agreement with Western blotting. For example, geometrically, the preferred interacting partner for CXCR4 M24 is CXCL12 S16 (and not CXCL12 E15), whereas the crosslinks between CXCR4 S9 and CXCL12 H17, S9 and L29, or G19 and K27 could not be reproduced in the best-scoring model (consistent with the majority of the crosslinks) at all. We therefore hypothesize that these crosslinks represent a transient interaction geometry which was captured strongly in native cell membranes by flow cytometry but was not stable as a purified crosslinked complex in detergent micelles for Western blot. The agreement of the model and pull-down Western blotting results demonstrate that our proposed method of flexible docking with local distance restraints efficiently resolves, in a fully unbiased manner, the small number of ambiguities and false positives produced by the flow cytometry screen of crosslinked complexes.

It may be worth revisiting page 12, line 272, but also page 13, lines 281 and following, taking into major account the WB data.

(***Clarification:*** *the reviewer refers to the following text:*

- *Page 12 line 272: The final full-length model places in close vicinity those residue pairs that were demonstrated to crosslink, whereas non-crosslinking residues are mostly distant (Supplementary Fig. 11).*
- *Page 13 line 281: Finally, several weakly positive receptor-chemokine residue pairs could not be explained by either our best scoring model geometry or by the alternative/transient geometries (Supplementary Fig. 12).*)

**Response:** We added the above reasoning to the manuscript text.

It would instead be interesting to have an overview of the experimental data against the distribution of the ligand-receptor distances measured in the final model.

**Response:** We refer the Reviewer to S11 Fig where we plot the receptor-ligand distances in the same heatmap representation as the experimental data shown in Fig 2. This serves the purpose suggested by the Reviewer. Additionally, we calculated the percentage of strongest crosslinks captured by our model at shortest distances, in comparison with other published models of the same complex (S13 Fig); again, this addresses the Reviewer's suggestion.

In any case, I recommend to soften the statement at page 10, lines 218-220.

(***Clarification*** *- the reviewer refers to the following text: "In general, detection of crosslinked complexes by flow cytometry proved sensitive and predictive of crosslinking in purified samples (Supplementary Fig. 4), suggesting that for future studies, flow cytometry detection alone will be sufficient to deduce the interaction geometry."*)

**Response:** In light of the comments by Reviewers #1 and #3 regarding the discrepancies between crosslinking efficiency by flow cytometry and Western blot, we have now included in the manuscript some of the discussion above, and softened our language around this paragraph as follows:

*"In general, crosslinked chemokine quantification by pull-down and Western blotting agreed well with that by flow cytometry, as indicated by their positive relationship (S4A Fig). This confirmed that flow cytometry is sufficiently sensitive and predictive of crosslinked complex detection in pulled down samples, while being much less labor-intensive. For future studies, flow cytometry detection alone should be sufficient to identify crosslinked receptor-chemokine complexes and inform efforts of deducing their interaction geometry by molecular modeling."*

Finally, authors validate the model by mutating charged residues of the receptor predicted to come into proximity of reversely charged residues in the ligand. Did the author also perform charge exchange experiments?

**Response:**
Following our discovery, in the original manuscript, of the detrimental effects of D20R and E26R mutations in CXCR4 CRS1, we here performed additional experiments seeking rescue of the signaling deficits by reciprocal charge reversals. Guided by the model, we cloned, expressed and purified chemokine mutants R47E and K56E. Combining CXCR4(E26R) with CXCL12(R47E) partially rescued functional potency and efficacy deficits caused by each mutation individually (Fig 5, included below), which confirms their direct interaction as predicted by the model. By contrast, combining CXCR4(D20R) with CXCL12(R47E) resulted in no rescue, because these residues do not interact directly (Fig. 5). For the second predicted salt bridge, that of CXCR4 D20 with CXCL12 K56, rescue experiments were not attempted, because unlike CXCR4(D20R), the chemokine mutant CXCL12(K56E) did not suffer any detectable functional defects. This indicates that some other chemokine residue in proximity of CXCR4 D20 may be more important than K56.

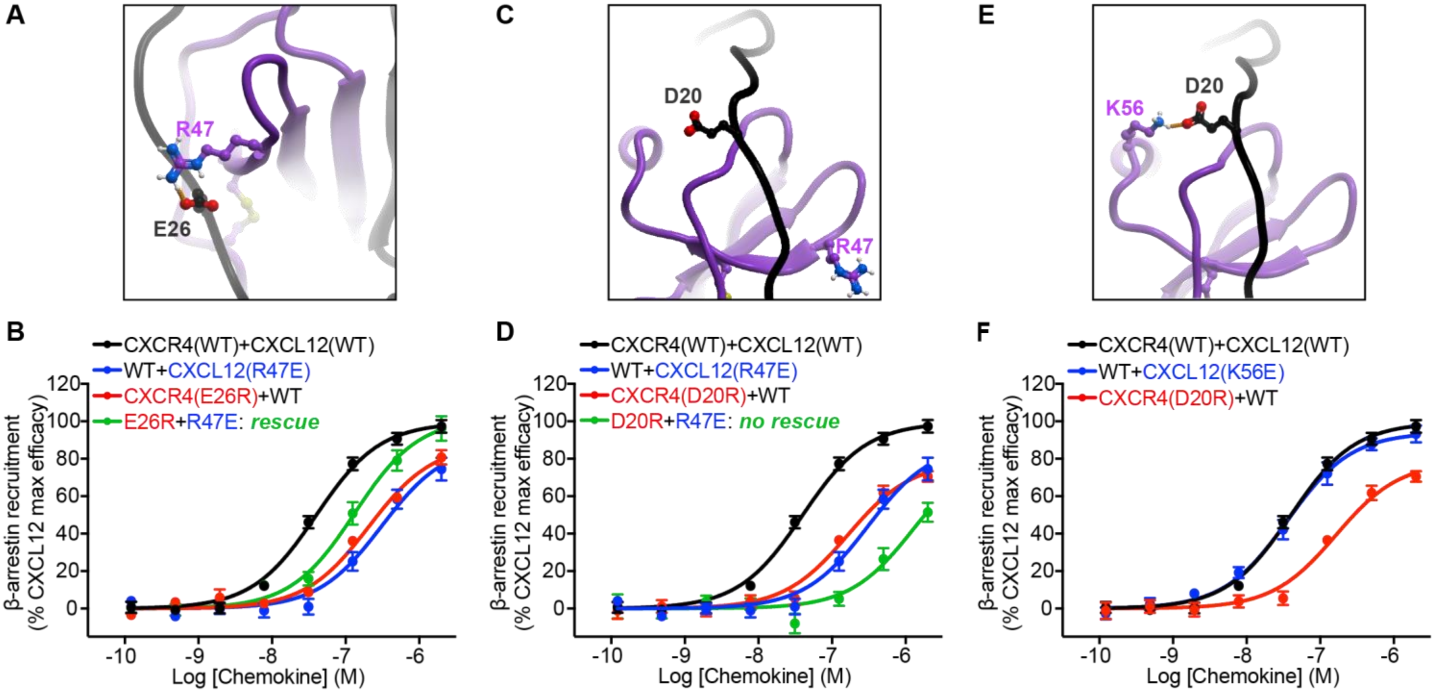

Overall, the paper is very interesting and relevant in the field. Molecular models based on crosslinking constraints are the best possible approximation when 3D structures are missing. Indeed, the model developed in this work reveals a number of interactions that were previously unknown and are nonetheless in line with previous functional studies. Therefore, the model is quite strong and it is very thoroughly discussed in the frame of existing literature in the discussion. Although the work is based on extensive and solid data, the handling of the data is in some places questionable, as discussed above. With respect to the scholar presentation, the manuscript indulges in a lot of details that make it difficult for the reader to gain a comprehensive overview of the story and make the overall manuscript very heavy. Moreover, there are parts in the experimental section that in fact belong to the discussion, for instance major sections of the paragraphs between lines 257-290. I am pretty confident that most of these issues can be resolved through a deep revision of the written text. This can become a really good paper.

**Response:** We have substantially modified the manuscript text and have improved the readability of the manuscript for a broader audience.

Minor issues

Page 7, line 133. A preliminary sentence providing a first overview of the different blocks of mutants that have been tested would help the reader to go through the following section.

**Response:** This suggestion is in line with similar suggestions made by other Reviewers about the presentation of the paper. We have now modified Figure 1 (panels J and K) so that it now provides a broad overview of the current knowledge of chemokine recognition sites. Adjacent to it, we have also included the same cartoon schematic used in Figure 2 to make the connection between Figure 1 and 2 a lot easier for the readers. As with the text, we have now modified it to refer to these schematics in Figure 1J and 1K, allowing the reader to easily visualize the different blocks of mutants that are being tested.

Page 7, line 135. Isn't here 30? In Fig. 2A I count 42 when controls are included.

**Response:** Yes, 30 is correct here - we have edited the manuscript.

Page 7, line 150-151. I find this sentence confusing, the aim is to say the occurrence and intensity of crosslinking was position-dependent, right?

**Response:** In the original submission, the sentence in question read ***"****Although the crosslinking pattern did not reveal a one-to-one correspondence between the receptor and chemokine residues, definitive asymmetry was observed".* What we mean here is that each residue in the distal N-terminus of the receptor demonstrated positive crosslinking signal not with one, but typically with several residues on the chemokine, and vice versa, each chemokine residue crosslinked with more than one receptor residue. In the ultimate case of such one-to-many crosslinks, each residue on one protein would crosslink equally well with **all** residues on the respective interface of the other protein, effectively obliterating any informative signal. However, this is not what we observed, as our crosslinking heatmap shows preferential positive crosslinking in three corners but not in the fourth, i.e. is not symmetric. We believe that the phrase "***not a one-to-one correspondence****, but* ***definitive asymmetry*** *is observed*" captures this idea succinctly. We interpret this observation as evidence of a particular (antiparallel) geometry in the CRS0.5, because if it were not the case, the crosslinking pattern would have been identical for the residues that flank the chemokine b1-strand or the residue in the receptor distal N-terminus. We have modified this section to make our logic clearer.

I could not find the information about the length of the distance restraints?

**Response:** Distance restraints were implemented using the mechanism in the ICM modeling software, where the restraints have no defined "length". Instead, the energy associated with a distance restraint is a smooth function of distance between the two atoms in question. Energies associated with the local distance restraints in this work are shown in S15 Fig; they are representative of our simulations, where the restraints were set between the Cβ atoms of cross-linked residues. As S15 Fig suggests, distances below 4Å are given minimum (most favorable) energy based on the weight; this is in line with Cβ-Cβ distances in disulfide bonds which average at ~3.9Å. For Cβ-Cβ distances exceeding 4Å, the energy gradually becomes weaker (less favorable) but it approaches an asymptote as the distance between the two atoms grows, rather than increasing indefinitely. This is a defining feature of **local** (as opposed to **global**) distance restraints. We have now included this additional information into the manuscript.

**Reviewer #2 critiques and responses**

Minor comment:

- Supplementary Table 4. Crosslinking efficiency is shown without any estimate of errors. That should be included as the data is clearly available (error bars are shown in Fig. 3c)

**Response:** We have rectified this oversight and now included the error in S4 Table.

**Reviewer #3 critiques and responses**

1) Overall, it is rather difficult to read. This could easily be improved by another round of editing with a view of the international audience that is the likely audience.

**Response:** In line with similar comments made by all Reviewers, we have improved the presentation and flow of the manuscript for a broader audience. In particular, we have made the text sections describing Figures 1 and 2 less dense and made the connections between Figures easier to follow as suggested by the Reviewer below.

2) The computational model on which authors base their selection of cross-linking pairs, is not very well presented in this manuscript. There is only a global view presented on Fig 1, and the details are missing. The Fig 1 of the accompanying manuscript is , on the other hand, a much better description of this model.

**Response:** Figure 1 of the present manuscript presents nothing more than a low-resolution hypothesis used to broadly guide the selection of residue groups for crosslinking. The high-resolution model that resulted from crosslinking-informed molecular docking is presented in Figure 4 of the present manuscript. Incidentally, this refined high-resolution model derived was also used as a framework for interpreting the mutagenesis experiments in Stephens et al., which explains why Stephens et al. Figure 1 presents it in greater detail.

To address the reviewer's comment, we have modified (now) Fig 1J-K to reflect our working hypothesis that the N-terminus of CXCR4 can be placed and interact with various grooves identified in our structural and physicochemical analysis of CXCL12. The text has also been modified to emphasize the black strokes drawn in Fig 1D-I, which highlight these grooves. Fig 1J and 1K also provides a broad overview of the different interfaces that guided our crosslinking studies:

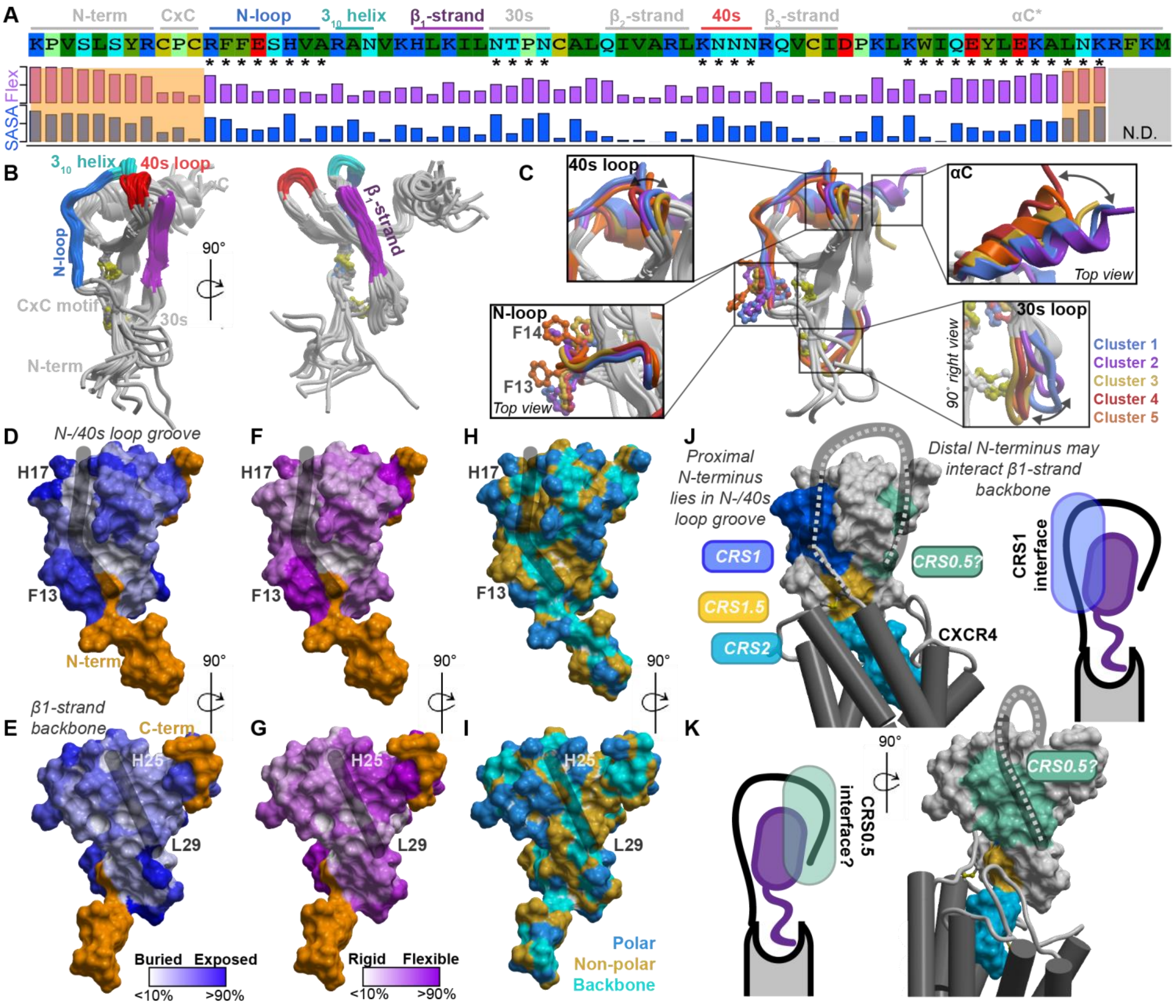


3) P16 l335-336 .. “we mapped, in a comprehensive and unbiased manner, pairwise residues…”. This should be discussed in depth as this type of mapping may not be as unbiased as one would think. It is based on a model that the authors have built initially to restrict the number of potential cross-linking pairs to be studied. Unfortunately, cross-linking is prone to stabilise some transient interactions. It is, effectively, searching for the lost keys under the lamppost, and may introduce a model bias through the “streetlight effect”. It would be important to highlight the cross-linking pairs or their absence that do confirm that the cross-linking is specific and is not influenced by the model. This, for example, could be argued by showing the data for a significant number of negative controls (comparable to the number of positive cross-links).

**Response:** We are very aware of the lamppost effect and made every effort to avoid it in our work in general and in this study in particular.

- First, it is important to note that the residue pairs for crosslinking were **not** selected based on a model. Instead, a model, or rather a low-resolution working hypothesis derived from property analysis of the chemokine alone (Fig 1), was used to delineate two broad **sites**, or **patches**, where interacting residues could be located. The first patch involved 9 residues from the chemokine and 10 residues from the receptor, whose crosslinking patterns were verified in the most unbiased manner possible by testing **each**

**against each** (90 pairs total, Fig 1J). The second patch involved 5 residues from the chemokine and 6 residues from the receptor, and was treated similarly (30 pairs total, Fig 1K).

- Next, 42 residue pairs were selected and tested **across** the predicted patches, serving as the negative controls for our hypothesis (Fig 2E and 2F). Interestingly, some of these negative control pairs demonstrated weak but detectable crosslinking, i.e. proved to be not quite negative. For example, the pair of CXCR4 S9 (CRS0.5) and CXCL12 H17 (CRS1, the N-loop of the chemokine) demonstrated a positive signal which is likely an example of crosslinking stabilizing a transient interaction, as suggested by the reviewer. We highlighted this transiently captured geometry in S12 Fig of the manuscript and have mentioned it on P12 l275-276 of the original manuscript. We also purified the S9C:L29C and K25C:A21C negative control pairs and detected negligible crosslinking when visualized by Western blot (Fig 4A).
- Finally, and accounting for these ambiguities, we included **all** positive crosslinks (>65% efficiency by flow cytometry, including control pairs spanning the two interfaces) in the docking simulation as local distance restraints, and allowed the unbiased conformational sampling procedure to find a conformation that satisfies as many of the restraints as possible, in the face of the experimentally detected ambiguities. No filtering or bias towards the starting hypothesis were introduced.

To continue with the unbiased analysis, we evaluated the resulting model in comparison with other published models against our set of experimental crosslinks, including the negative controls (S11 Fig and S13 Fig). As expected, our model **guided** by the crosslinks was also **most consistent** with the crosslinks. Among other findings, our each-to-each crosslinking effort in the hypothetical CRS0.5 clearly distinguished the anti-parallel β-sheet interaction between the distal N-terminus of CXCR4 and the CXCL12 β1-strand, as compared to the parallel geometry proposed by others (Ziarek et al).

In summary, and based on these considerations, we believe that the sentence in the manuscript was accurate. For clarity, we added highlights of these considerations to the manuscript in the 2nd section of Results (*Flow cytometry-based disulfide crosslinking identifies prominent CXCR4-CXCL12 residue proximities along the predicted interaction sites*).

How different was the final experimental-results constrained model to the initial prediction?

**Response:** As explained in the response to Comment #2 above, the initial "prediction" was nothing more than a structural hypothesis outlining the broad patches on the chemokine where the different segments of the CXCR4 N-terminus may interact. The final model is consistent with this initial hypothesis, i.e. the segments of the CXCR4 N-terminus do indeed interact with those patches on the chemokine that we predicted initially. However, geometric comparisons (e.g. RMSD) are not possible, for the lack of defined geometry in the initial hypothesis. This is now clarified in the manuscript.

4) As is clear from figure 3b/c and S4 that there are some significant disagreements between the flow cytometry and the western blot data. What is the correlation coefficient between the data? Could the authors explain what are the reasons for these differences? Could the authors elaborate why one experiment would provide better accuracy than the other? The authors seem to assume the western blot data is more reliable: what is the agreement between the final model and the western blot cross linking data (eg, fig S9 and S11?).

**Response:** We refer to our response to Reviewer 1 Comment #3 where we explain why our original method of quantifying Western blot crosslinking efficiency produced results that were poorly relatable to flow cytometry and as a result, hard to interpret. In the course of the revision, in light of the Reviewer 1 and 3 comments, we modified our approach to quantifying the Western blot data and separated the two factors in this quantification that were initially multiplied to obtain the final number. This made the newly quantified Western blot-based data directly

comparable to the flow cytometry data. The crosslinking efficiencies by flow cytometry and Western blot are now consistent with a slope of 1.1 and a correlation R = 0.69.

Pull-down and Western blotting may or may not be a more reliable technique for crosslink quantification; but it is definitely a more traditional experimental approach [Qin et al., Science 2015, Kufareva et al., Methods Enzymol 2016, Gustavsson et al., NComms 2017]. Because our manuscript is the first study to propose and optimize the new flow cytometry approach, and to systematically evaluate it for the detection of crosslinked complexes, we chose to independently verify the results by an orthogonal and established method, to build confidence in the robustness of the new approach. Additionally, pull-downs and Western blotting can provide extra information about the crosslinked complexes, that is not accessible by flow cytometry, such as the fraction of extracted receptor that is crosslinked. Overall, the two approaches are prone to different sets of caveats; despite this, the positive relationship between their results suggests that the newly developed flow cytometry approach is predictive of crosslinking in pulled down samples.

This said, several points remained discordant (see our response to Reviewer 1 above), with flow cytometry quantification providing a positive result whereas pull-down and Western blotting suggested weak or no crosslinking. For these pairs, even though the positive flow-cytometry-based crosslinks were present in the modeling procedure as distance restraints, the final result of modeling tends to agree with Western blotting. This indicates that compared to flow cytometry, Western blotting may be less tolerant to suboptimal complex geometries. However, it also demonstrates that by virtue of higher throughput, flow cytometry can efficiently inform molecular modeling efforts, and that complementing flow cytometry by modeling can robustly resolve the small number of experimental ambiguities and false positives.

Minor issues:

1) The authors claim in the abstract to have found the basis for chemokine selectivity that is caused by an alternative positioning of the N-terminus of the chemokine in the TM bundle of the receptor. It is worth explaining in the abstract how this is determined by the binding mode of the N-terminus of the receptor (eg, rotational positioning of the chemokine molecule relative to the receptor).

**Response:** The complete receptor-chemokine complex model presented here is a result of several advances, including (i) definitive, experimentally guided interaction geometry in CRS0.5 and CRS1, (ii) refined geometry in CRS0.5, (iii) improved positioning of the globular core of the chemokine w.r.t. the TM bundle of the receptor, and (iv) improved interhelical packing in the receptor. Together, these advances enabled the *ab initio* prediction of the conformation of CXCL12 N-terminus that proved consistent with CXC-specific geometric constraints and prior mutagenesis experiments, as well as the new findings in the accompanying manuscript by Stephens et al.; moreover, it is this (previously unobserved) conformation of the chemokine N-terminus that provides a natural explanation for the question of chemokine selectivity. The abstract has been modified accordingly.

2) p5-6 Several characteristics of the CXCL12 structure are listed. The readability would greatly improve if the relevant subfigures of figure 1 are mentioned for each bullet point.

**Response:** The relevant subfigures of Fig 1 have now been mentioned for each bullet point.

3) On p8, line 159-160, the authors reference figure 1, but it is not clear from figure 1 what “the proposed structural role of L26 and I28” is.

**Response:** Residues L26 and I28 likely have a structural role in forming the β-sheet motif in the chemokine β1-strand. We have clarified this sentence in the text.

4) P10 l224 Wrong reference: Fig S1 does not contain a model of the CXCR4-CXCL12 complex.

**Response:** We are referring to our study workflow here.

5) P24 l547: The formula contains the word "ratio". This might be confusing, since the ratio has already been written out (by the "/" character). What software was used to analyse the Western blots and the SDS-page densitometry? What bands were taken into account for the densitometry reading? How does this give more information that IR800/IR680?

**Response:** ImageStudioLite was used for quantification of both Western blot and SDS-PAGE.

In the revised manuscript, to quantify the amount of crosslinked chemokine, a rectangle was drawn on the anti-HA Western blot that captures the crosslinked chemokine band, and signal intensity within the rectangle was recorded as reported by ImageStudioLite. This value was used as the crosslinking efficiency by Western blot. To calculate the percentage of crosslinked receptor, the same chemokine rectangle was used to capture the upper chemokine-crosslinked receptor band, and a second rectangle was drawn to capture the total receptor band. The percent crosslinked receptor was determined by: upper band IR680 signal/total receptor IR680, where IR680 reports the Flag tag on the receptor. The area of the rectangles was kept consistent for all samples within a single Western blot.

This quantification method is slightly different from that presented in the original manuscript. Specifically, we separated the two pieces of information provided by the Western blot experiments: amount of chemokine and fraction of the extracted receptor that is crosslinked. We modified the Methods appropriately to reflect this, and also included all of these values in S4 Table. The change was introduced to address Reviewer 1 Comment #3 and this Reviewer's Comment #3 above. We refer to our response to Reviewer 1 Comment #3 above to explain our reasoning.

6) P25 l563: The authors use "weighted local harmonic distance restraints" for their model. Is this based on both the flow cytometry data and/or the western blot data? If both, how were they combined?

**Response:** We apologize for the confusion here. In fact, our distance restraints weights were based only on the receptor-chemokine pairs which demonstrated >65% crosslinking efficiency as determined by flow cytometry. As explained above in response to Reviewer 1 comment #3, Western blot data was not used to determine the weights, to ensure that our crosslinking restraint set is large, unbiased, and methodologically uniform. We have now added the weight of each respective distance restraint to S4 Table.

7) Fig. 2: Please add explanation of the grey squares in the heatmap. Intuitively, one would interpret is a "normal" or 100% of the reference, but 100% is shown in red.

**Response:** Grey squares in the heat map represent residue pairs that were not studied. We have added this information to the Figure 2 legend.

**Reviewer #4 critiques and responses**

(1) The study would benefit from demonstrating that for the top cross-linking cysteine couples, computational models can be obtained wherein the two residues are mutated to cysteines which approach each other within disulfide-bonding distances. Such structural analysis is important to provide a true structural context for the cross-linking approach.

**Response:** As suggested by the Reviewer, we have now added an extra Supplementary Figure (S9 Fig) and modified the manuscript accordingly, presenting a model of CXCR4-CXCL12 with two explicit disulfides (K25C:E15C and G3C:L29C) which provide a structural context to our crosslinking approach. The disulfides can easily be accommodated in our top ranking model with minor changes to the receptor N-terminus conformation.

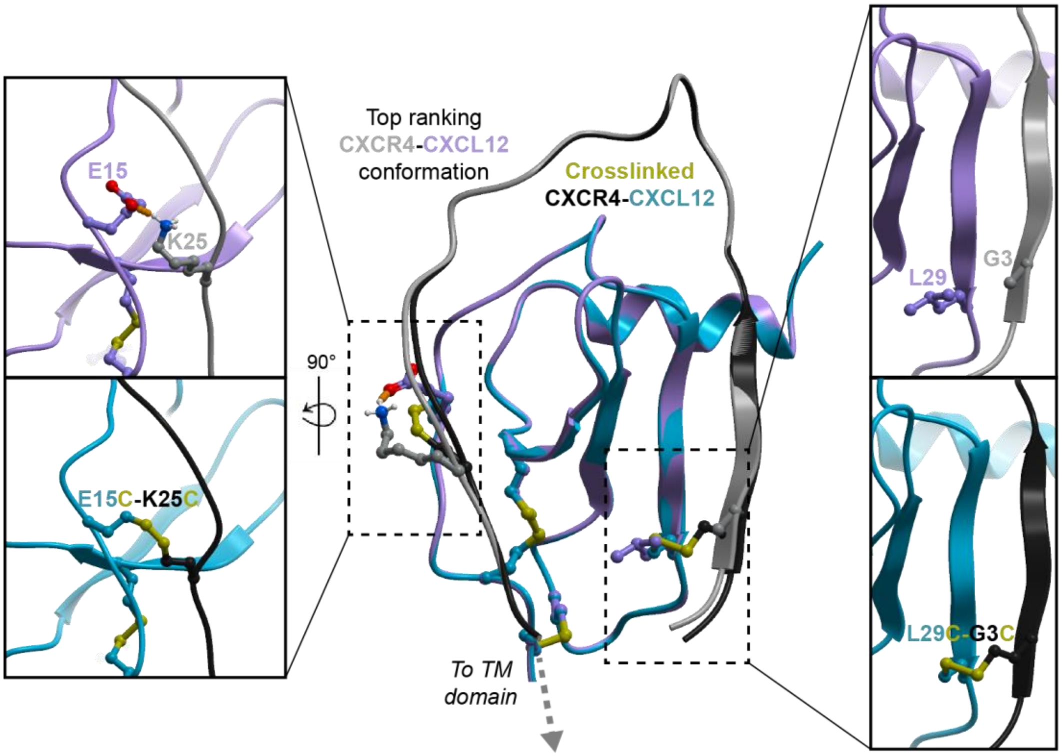


(2) The readability of the manuscript could benefit from a structural model, preferably in schematic mode, that summarizes the current understanding of CXC receptor-chemokine assemblies in terms of the site 1/site 2 models.

**Response:** This comment was similarly made by the other Reviewers. To address these comments, we have now modified Figure 1 (panels J and K) that summarizes our current knowledge of chemokine recognition sites. Adjacent to it, we have also included the same cartoon schematic used in Figure 2 to make the connection between Figure 1 and 2 easier for the readers.

Additional comments to improve display items:

-The electrostatic potential scale used in Fig. 1g for CXCL12 basically suggests that the entire face shown is covered with positively charged residues. This cannot be correct. The authors will need to specify the electrostatic potential scale shown and how this calculation was done.

**Response:** In fact, the visual impression created by S2 Fig (formerly Fig 1g) is accurate. Many chemokines are strongly positively charged molecules, and CXCL12 is no exception. In CXCL12, there are only four negatively charged residues, compared to the total of seventeen positively charged residues, including two pH-dependent His residues (Fig 1A). The image in S2 Fig was generated using macro dsRebel in ICM [Abagyan et al., J Comp Chem 1994] which colors the molecular surface according to the electrostatic potential calculated by the REBEL (Rapid Exact-Boundary ELectrostatics) method [Totrov & Abagyan, Biopolymers 2001]. REBEL solves the Poisson equation using the analytical molecular surface as dielectric boundary. The parameters were set at their

default values: maxColorPotential = 5. and TOOLS.rebelPatchSize = 1., and hydrogen atoms were ignored (as is the default).

-Supplementary Table 2 is lacking units of Å2 for the B-factors.
-Supplementary Table 3 is lacking units of Å for the resolution column.

**Response:** We thank the reviewer for pointing it out. Corresponding changes have been made to the manuscript.

-Several of the figures feature color combinations that are difficult to discern. For instance, Supplementary Fig. 6 combines purple with black for the chemokine and receptor, respectively.

**Response:** We have now increased the size and lightened the purple shade of the chemokine which has clarified the details of the receptor-chemokine interactions shown in (now) S7 Fig.
